# Genomic selection reveals hidden relatedness and increased breeding efficiency in western redcedar polycross breeding

**DOI:** 10.1111/eva.13463

**Published:** 2022-08-23

**Authors:** Omnia Gamal El‐Dien, Tal J. Shalev, Macaire M. S. Yuen, Rod Stirling, Lori D. Daniels, Jesse W. Breinholt, Leandro G. Neves, Matias Kirst, Lise Van der Merwe, Alvin D. Yanchuk, Carol Ritland, John H. Russell, Joerg Bohlmann

**Affiliations:** ^1^ Michael Smith Laboratories University of British Columbia Vancouver British Columbia Canada; ^2^ Pharmacognosy Department, Faculty of Pharmacy Alexandria University Alexandria Egypt; ^3^ FPInnovations Vancouver British Columbia Canada; ^4^ Department of Forest and Conservation Sciences University of British Columbia Vancouver British Columbia Canada; ^5^ Rapid Genomics Gainesville Florida USA; ^6^ Intermountain Healthcare Intermountain Precision Genomics St. George Utah USA; ^7^ School of Forest, Fisheries and Geomatic Sciences University of Florida Gainesville Florida USA; ^8^ British Columbia Ministry of Forests Lands and Natural Resource Operations and Rural Development Victoria British Columbia Canada; ^9^ Department of Botany University of British Columbia Vancouver British Columbia Canada

**Keywords:** accuracy, breeding, conifers, GBLUP, genetic gain, genomic selection, pedigree reconstruction, polycross, resistance, western redcedar

## Abstract

Western redcedar (WRC) is an ecologically and economically important forest tree species characterized by low genetic diversity with high self‐compatibility and high heartwood durability. Using sequence capture genotyping of target genic and non‐genic regions, we genotyped 44 parent trees and 1520 offspring trees representing 26 polycross (PX) families collected from three progeny test sites using 45,378 SNPs. Trees were phenotyped for eight traits related to growth, heartwood and foliar chemistry associated with wood durability and deer browse resistance. We used the *genomic realized relationship matrix* for paternity assignment, maternal pedigree correction, and to estimate genetic parameters. We compared genomics‐based (GBLUP) and two pedigree‐based (ABLUP: polycross and reconstructed full‐sib [FS] pedigrees) models. Models were extended to estimate dominance genetic effects. Pedigree reconstruction revealed significant unequal male contribution and separated the 26 PX families into 438 FS families. Traditional maternal PX pedigree analysis resulted in up to 51% overestimation in genetic gain and 44% in diversity. Genomic analysis resulted in up to 22% improvement in offspring breeding value (BV) theoretical accuracy, 35% increase in expected genetic gain for forward selection, and doubled selection intensity for backward selection. Overall, all traits showed low to moderate heritability (0.09–0.28), moderate genotype by environment interaction (type‐B genetic correlation: 0.51–0.80), low to high expected genetic gain (6.01%–55%), and no significant negative genetic correlation reflecting no large trade‐offs for multi‐trait selection. Only three traits showed a significant dominance effect. GBLUP resulted in smaller but more accurate heritability estimates for five traits, but larger estimates for the wood traits. Comparison between all, genic‐coding, genic‐non‐coding and intergenic SNPs showed little difference in genetic estimates. In summary, we show that GBLUP overcomes the PX limitations, successfully captures expected historical and hidden relatedness as well as linkage disequilibrium (LD), and results in increased breeding efficiency in WRC.

## 1 INTRODUCTION

Western redcedar (WRC, *Thuja plicata* Donn *ex* Don, Cupressaceae), is a conifer native to the Pacific Northwest of North America. WRC is a slow‐growing species that can typically grow to a height of 60 m and diameters ranging from 150 to 300 cm on a wide variety of sites depending on light availability, elevations, soils, and moisture levels (Minore, 1983). The species' tolerance to biotic and abiotic stresses and its remarkable phenotypic plasticity are likely to have contributed to its range of distribution (Antos et al., 2016; El‐Kassaby, 1999). WRC is one of the most economically important conifers on the west coast of Canada, with up to 10 million seedlings planted in British Columbia (BC) per year (Russell & Daniels, 2010), and is an essential cultural resource for Indigenous peoples of the region (Benner et al., 2021; Zahn et al., 2018). WRC is known for its natural wood durability, securing an international economic niche for outdoor wood products (Stirling et al., 2017). A WRC breeding program in BC is developing advanced breeding populations that are resilient to foliar pathogens and deer browsing, while improving wood durability and growth (Russell & Yanchuk, 2012). Climate change is expected to worsen WRC susceptibility to pests and pathogens impacting tree survival and adaptability (Berg et al., 2006; Gauthier et al., 2014; Sturrock et al., 2011). However, climate models also predict the range of WRC to expand over the next century, especially in the interior of BC (Hamann & Wang, 2006).

Several specialized secondary metabolites in foliage and heartwood are reported to be responsible for the resilience of WRC at different life stages. Ungulate browsing is a major risk factor affecting WRC seedling survival (Russell, 2008; Vourc'h, De Garine‐Wichatitsky et al., 2002; Vourc'h, Russell, & Martin, 2002). Trees with higher foliar monoterpene levels are significantly less browsed by deer than those with lower levels (Kimball et al., 2012; Russell, 2008; Vourc'h, De Garine‐Wichatitsky et al., 2002; Vourc'h, Russell, & Martin, 2002). Heartwood decay is frequently found in mature trees and impacts recovery and utilization of wood products (Buckland, 1946; Van Der Kamp, 1975). Two main chemical classes of heartwood extractives, thujaplicins (cyclic terpenoids derived from tropolones) and lignans, are associated with heartwood durability and rot resistance (DeBell et al., 1997; Stirling & Morris, 2016). Foliar terpenes are already present in young seedlings, and one‐year‐old plants can be selected for deer browse resistance using foliar terpenes. In contrast, heartwood chemicals are a mature trait that is not expressed until trees are at least around 20 years old, with lignans typically expressed at a later age compared to thujaplicins (Russell & Daniels, 2010). Genomic selection (GS) provides an opportunity to achieve earlier gains in wood durability traits, thus increasing the rate of genetic gain of a complex late‐expressed trait per unit time and cost (Grattapaglia, 2014; Isik et al., 2015). Selection for volume, growth and most other traits of interest in the WRC breeding program are typically assessed between years 7 and 10 (Russell & Yanchuk, 2012).

Compared to breeding programs for other conifers, WRC breeding benefits from several unique biological features of the species; specifically, WRC is a self‐compatible species, flowering and reproduction can be induced in WRC seedlings at less than one year of age, and self‐compatibility in WRC is accompanied with extremely low inbreeding depression for growth traits (Russell et al., 2003; Russell & Ferguson, 2008; Wang & Russel, 2006). Notably, WRC appears to have limited genetic variation compared to other conifers (Copes, 1981; Critchfield, 1984; El‐Kassaby, 1991; El‐Kassaby et al., 1994; Glaubitz et al., 2000; Yeh, 1988). Microsatellite markers suggest that all current WRC populations originated from a single refugium at the southern end of its current range of distribution (O'Connell et al., 2008), which leads us to hypothesize that there is hidden relatedness in the current populations. Limited genetic variation has been confirmed recently using single‐nucleotide polymorphic (SNP) data (T. Shalev, O. Gamal El‐Dien, M. M. S. Yuen, L. Van der Merwe, J. W. Breinholt, L. G. Neves, M. Kirst, A. D. Yanchuk, C. Ritland, J. H. Russell, J. Bohlmann, unpublished data). However, several recent studies revealed some quantitative variation in certain fitness traits such as growth, foliar monoterpenes, cedar leaf blight (CLB) resistance, and cold hardiness (Cherry, 1995; Rehfeldt, 1994; Russell & Yanchuk, 2012; Vourc'h, De Garine‐Wichatitsky et al., 2002; Vourc'h, Russell, & Martin, 2002).

The polycross (PX) mating design is one of the most cost‐effective breeding schemes for forest trees, in which a female parent tree (FPT) is pollinated with a pollen mix collected from a group of known males (Kumar et al., 2007). This mating design has traditionally been used to screen a large number of FPTs for backward selection, but has recently gained interest for forward selection after pedigree reconstruction (Lenz, Nadeau, Azaiez et al., 2020; Vidal et al., 2015, 2017). PX is preferred over open pollinated (OP) mating design in WRC to reduce selfing, as it occurs in open‐pollinated conditions (O'Connell et al., 2004; Russell & Yanchuk, 2012). However, PX may result in overestimation of genetic parameters when not meeting the assumptions of being true half‐sibs (HS) within the maternal family with an equal male contribution in the pollination process (El‐Kassaby et al., 2011; Vidal et al., 2015).

Given the assumed limited genetic variation in WRC and self‐compatibility, it is expected that the species has a high level of historical relatedness and high linkage disequilibrium (LD), i.e., non‐random associations of alleles, compared to other conifers (T. Shalev, O. Gamal El‐Dien, M. M. S. Yuen, L. Van der Merwe, J. W. Breinholt, L. G. Neves, M. Kirst, A. D. Yanchuk, C. Ritland, J. H. Russell, J. Bohlmann, unpublished data). However, the traditional breeding approach in WRC, using pedigree information, cannot capture these two features. Molecular breeding and the use of Genomic‐based Best Linear Unbiased Prediction analysis (GBLUP) may provide strategies for increasing breeding efficiency and genetic gains. GBLUP will also correct for any violations from the maternal HS relationship, and the equal male contribution assumptions of the PX mating design, by using the estimated actual relationship between individuals. GS, which predicts phenotypes from genomic data (Meuwissen et al., 2001), coupled with the induction of early age flowering in WRC (Russell & Ferguson, 2008), has the potential to reduce the WRC breeding cycle from 20–25 to 2–4 years. This advanced breeding strategy may be the most efficient for the improvement of WRC high‐value wood traits.

The outperformance of GBLUP over traditional pedigree‐based BLUP analysis (ABLUP) has been reported in several studies (Beaulieu et al., 2020; Calleja‐Rodriguez et al., 2020; El‐Kassaby et al., 2020; Lenz, Nadeau, Mottet et al., 2020; Ratcliffe et al., 2015). In PX and OP mating design, GBLUP has the added advantage of estimating the non‐additive effect over pedigree models, resulting in better separation of additive and non‐additive effects, which can lead to more accurate estimates of BVs (Gamal El‐Dien et al., 2016, 2018; Lenz, Nadeau, Azaiez et al., 2020). Given the expected larger paternal HS families in PX compared to OP, due to the use of a group of known males, PX will benefit the most from pedigree reconstruction or GBLUP compared to all other mating designs, such as full‐sib (FS) and OP.

Genomic selection has also brought two major benefits to breeding, increased breeding value (BV) accuracy (when the phenotype is available) and prediction of BV at a younger age for early selection (when the phenotype is absent). In this study, we focus on the first aspect paving the way for the second. Here, we present the use of GBLUP in a WRC breeding program to detect hidden historical relatedness, capture LD, and overcome weaknesses of the PX mating design for forward selections by selecting superior recombinant offspring in maternal families. The specific objectives of the current study were: (1) Apply the ***G*** matrix to paternity assignment and pedigree correction, converting the PX‐ to an FS‐pedigree; (2) examine deviation from the PX assumption of HS relationship and the equal male contribution, and the resulting inflation in genetic estimates; (3) use GBLUP to estimate BVs, heritability, genotype × environment (G × E), dominance effects, and genetic correlations between all traits; (4) quantify the improvement of GBLUP in BV prediction accuracy and expected genetic gain over the PX‐ and FS‐pedigrees; and (5) compare the use of different types and numbers of SNPs (all SNPs versus genic‐coding, genic‐non‐coding, intergenic SNPs only) and their effect on heritability.

## 2 MATERIAL AND METHODS

### 2.1 Plant materials

**FIGURE 1:**
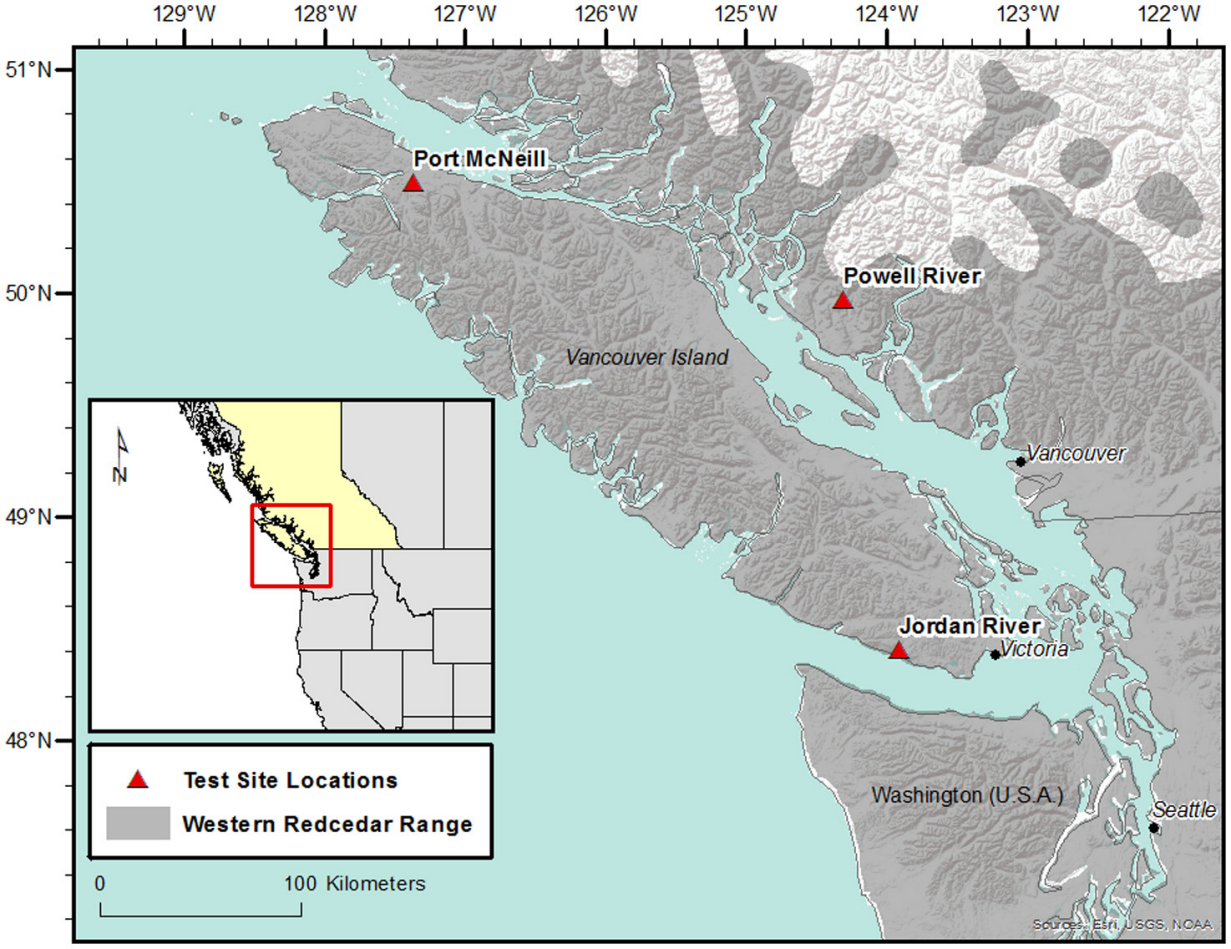
The western redcedar range and the location of the three polycross progeny tests in the province of British Columbia, Canada. The test sites are located at Jordan River, Port McNeill and Powell River. The climate variables (annual temperature, mean annual precipitation, heat moisture index, and degree days over 0 degrees) for the three sites are: Jordan River (7.7, 2480 mm, 7.1, and 168); Port McNeill (8.2, 2302 mm, 7.9, and 135); and Powell River (9.2, 1400 mm, 13.7, and 132).

We sampled a 1st generation PX progeny trial established by the BC Ministry of Forests, Lands and Natural Resource Operations and Rural Development. A total of 1000 parent trees were polycrossed with a common set of pollen parents, over an eight‐year period, resulting in seven testing series with four to seven field test sites per series. The 21 males used in the polymix were randomly selected, and the pollen was mixed in unequal proportions to consider different estimates of pollen germination rate (Russell & Yanchuk, 2012). The field design comprised single‐tree plots with incomplete block design. Each field site included 35 replicates (blocks) with various numbers of sets (incomplete blocks within replicates) and sizes depending on the number of families within each set. For this study, we sampled trees from series #3, which tested 111 PX families for growth over six sites. Seedlings were one‐year old at planting in 2000. A total of 1520 offspring trees (OTs), representing 26 PX families, growing on three progeny test sites in Maritime Low (ML) Seed Planning Unit (the main geographic seed zone for WRC at the time of planting) were selected for this study. The three test sites are located near Jordan River (Lat. 48.442931°N; Long. 124.025437°W; Elev. 335 m), Port McNeill (Lat. 50.546111°N; Long. 127.340833°W; Elev. 150 m), and Powell River (Lat. 50.015756°N; Long: 124.347394°W; Elev. 140 m) (Figure 1). The 26 PX families used in our study were not randomly selected from the 111 PX families in the full trial. Out of the 26 PX families, 19 families were selected for high growth performance (high female parent BVs for height), six families for wood resistance (high female parent's thujaplicin concentration) and one family for foliar total monoterpene (high female parent BVs for foliar total monoterpene). We used all the 1520 offspring (26 PX families) in all analyses, regardless of the type of selection of their maternal parents. On average, 20 OTs/PX family were selected from each site. The numbers of OTs/PX family ranged from 38 to 61, with an average of 58 trees.

### 2.2 Phenotyping

#### 2.2.1 Growth traits

Tree height (HT, measured in cm) and tree diameter at breast height (DBH, measured in mm) were assessed at age 15. All trees from Jordan River and Port McNeill were found to be naturally infected with CLB, which significantly reduced the growth on these two sites (Figures S1 and S2). CLB infection was measured on a scale of 0–4 representing the percentage of infected foliage; where a 0, 1, 2, 3, and 4 score reflected <5%, 5%–25%, 26%–50%, 51%–75%, and 76%–100% of foliage, respectively. Based on this subjective measure, more than 85% of trees at the two sites had a score of 2 (26%–50% infected foliage). With a score of 2, a tree was visibly impacted by CLB in the lower, shaded foliage but showed minimal blight in the upper crown and continued to grow.

#### 2.2.2 Foliar chemical traits

For foliar monoterpene measurements, current year growth foliage was collected at age 15 and terpenes were quantified at the Glynn Road Analytical Chemistry Lab. Sample extraction, qualitative and quantitative analyses followed the method of Kimball et al. (2005) with some modifications. Approximately 250 mg (fresh weight) of frozen samples was ground and extracted into 4 ml of methanol containing pentadecane as an internal standard. Extracts were analyzed on a Clarus 580 gas chromatography (GC) using a 30M ZB‐5MSi GC column and flame ionization detector (FID). Peaks were identified using retention times comparison to reference standards, and GC/mass spectrometry (MS) was used for further confirmation as needed. Each foliage sample was measured for moisture content, and terpene contents were calculated based on a 70°C oven dry weight. Thirty‐two monoterpenes were identified and quantified using internal standard (pentadecane) and reported in μg/g dry foliar weight (DFW). Total monoterpene content was quantified by integrating all monoterpene peaks. We analyzed “foliar total monoterpenes, F.TM” as one variable summarizing all foliar monoterpenes to represent deer browsing resistance; we also analyzed “foliar α‐thujone, F.AT” as the major monoterpene in the foliar extract.

#### 2.2.3 Wood traits

At tree age 18 years, increment cores were sampled at breast height (1.3 m) from 1510 trees. Trees were cored three years after sampling of foliage, allowing for a longer time for the expected later expression of wood thujaplicin and lignan compared to growth and foliar traits. Each core represented the full diameter of the tree, intercepting the pith whenever possible, yielding one radius for dendrochronological analysis and the second radius for heartwood extractives analysis. The first radius of each core was dried and sanded to reveal individual cells, scanned at high resolution (1200 DPI), and the annual rings from the bark to pith were visually cross‐dated and counted (Stokes & Smiley, 1996). For the subset of 414 cores that did not intercept the pith, the number of missed rings (1.3 ± 0.6 rings) was estimated geometrically (Duncan, 1989) and added to the ring counts to represent the total number of rings. On the second radius of each core, the heartwood‐sapwood boundary was determined by qualitatively assessing the change in color of the xylem and the width of the heartwood from the boundary to the pith was measured to the nearest mm. Counting from the heartwood‐sapwood boundary toward the pith, five rings of heartwood were cut from the radius for processing for heartwood extractive analysis. If samples had fewer than five rings of heartwood, all available heartwood rings were processed, and the number of rings was documented. Samples were extracted and analyzed by high‐performance liquid chromatography (HPLC) using diode array detection according to Daniels and Russell (2007) with minor modifications to the gradient elution. Samples were analyzed, using an internal standard (crotonic acid para‐bromophenacyl ester in ethanol), on an Agilent 1260 system. Extracts were analyzed for seven known compounds using reference standards (plicatic acid, α‐, β‐, γ‐thujaplicin, β‐thujaplicinol, thujic acid and methyl thujate) and reported in μg/g conditioned wood weight (CWW), which is oven‐dried wood at 40°C for 48 h to avoid potential extractive loss due to volatilization. Peak area ratios/g CWW were calculated for 11 other compounds for which reference standards were not available. One of these compounds previously reported as compound B, was identified as plicatin (plicatic acid lactone) (Stirling & Morris, 2016). In this study, we used the sum of α‐, β‐, γ‐thujaplicin, and β‐thujaplicinol as a measure for total effective thujaplicins, “wood total thujaplicins, W.TT”; we also analyzed “wood α‐thujaplicin, W.AT” as the major thujaplicin in the extract. Combined analysis of the thujaplicins is based on their common biosynthetic pathways and their similarly high fungal inhibition (Rennerfelt, 1948; Roff & Whittaker, 1959). Both plicatic acid and plicatin are moderately correlated with decay resistance in wood (Morris & Stirling, 2012), but inhibit fungal growth much less than the thujaplicins in vitro (Roff & Atkinson, 1954). Plicatic acid was only detectable in 32% of our population, likely due to the relatively young age at sample collection time, while plicatin was detected in more than 95% of the samples. Here, we used the sum of these two compounds to represent total effective lignans, “wood total lignans, W.TL”; we also used “wood total extractives, W.TE”, which is the sum of the six effective compounds (the four thujaplicins and two lignans) as one trait representing heartwood extractives.

Only wood traits showed a non‐normal distribution; thus, log transformation was used to meet the assumption of normality (Figures S1 and S2). The mean, standard deviation (SD), and coefficient of variation (CV) for all traits are reported in Table S1. Box and density plots across the three sites were used to visualize within and between site variations for all traits (Figures S1 and S2).

### 2.3 Genotyping

Foliage samples were collected for DNA extraction, from the 1520 PX OTs at age 15, and from the 44 parent trees (representing the 26 female parent trees (FPTs) and the 21 PX male parent trees (MPTs), note, two parent trees were used as both female and male, and one female did not survive, which reduced the number of genotyped parent trees from 47 to 44). For sequencing of SNPs, we used 0.5 g of tissue from each sample which was lyophilized for 48 h prior to DNA extraction. DNA was isolated according to Xin and Chen (2012). The concentration and quality of DNA was verified with a Nanodrop 2000c (Fisher Scientific), Quantiflor (Promega Corporate) and 0.8% agarose gel to crosscheck for the quantity and quality of the DNA prior to submission to RAPiD Genomics LLC for genotyping.

For sequence capture genotyping, 20,858 probes designed from genic and non‐genic regions were used to analyze the 44 parents and 1520 OTs, as described in (T. Shalev, O. Gamal El‐Dien, M. M. S. Yuen, L. Van der Merwe, J. W. Breinholt, L. G. Neves, M. Kirst, A. D. Yanchuk, C. Ritland, J. H. Russell, J. Bohlmann, unpublished data). After initial biallelic SNP filtering for quality (minimum quality score of 10), missing data (sites with more than 40% missing data were removed), depth (minimum depth of 3, minimum mean depth of 15, maximum mean depth of 750), and minor allele frequency (MAF of 0.005), hence, 51,638 SNPs were chosen with average mean depth of 31×. Reproducibility was assessed by genotyping replicated control samples and estimated to be between 91% and 96%. The 51,638 SNPs were further filtered using a minimum quality score of 30, MAF of 0.01, maximum mean depth of 90, and sites with more than 20% missing data were removed using vcftools v0.1.17 (Danecek et al., 2011). SNPs with an allele balance (AB) >0.2 and lower than 0.8, or with AB close to zero were retained using vcffilter in vcflib (Garrison, 2016). Ten of the 1520 OTs with more than 40% missing data were removed. After filtering, all 44 parents, 1510 OTs, and the final 45,378 quality SNPs were retained for subsequent analyses. The percentage of missing genotypic data was 0.27%, and the expectation‐maximization (EM) imputation algorithm was used to impute the missing data using the R function “A.mat” in the R package “rrBLUP” (Endelman, 2011).

### 2.4 Pedigree verification, paternity assignment, and relationship matrices

The realized additive genomic relationship matrix (***G*** matrix) was constructed using 45,378 SNPs (***G***_all_) with the “A.mat” function in the R package “rrBLUP” (Endelman, 2011; Endelman & Jannink, 2012) for the 44 parents and 1510 OTs. The ‘default option’ was used in “rrBLUP”, which is equivalent to the formula described by VanRaden (2008). The relationship coefficients from the ***G*** matrix were then used to: (1) correct any error in the female assignment by using the normality properties of the realized relationship coefficient as proposed by Munoz et al. (2014) and investigate the peak at zero relationship within each family; (2) assign males; and (3) identify individuals resulting from selfing, mislabeled and duplicate genotyped samples. We first verified the maternal parent by looking into the FPT‐offspring and offspring‐offspring relationships within each maternal PX‐family. The relationship between each offspring and all FPTs was used to correct any error in the pedigree FPT by identifying the FPT showing the maximum relationship. We also examined the relationship within each maternal HS‐family and investigated the peak at 0 relationships (Figures S3 and S4). For the paternity assignment, the relationship between each offspring and all MPTs was used to assign the male parent by identifying the MPT showing the maximum relationship. Then, we confirmed all MPT assignments from the offspring‐offspring relationship within each paternal HS‐family. We used a relationship threshold of 0.15 and 0.07 for the parent‐offspring and HS offspring‐offspring relationship, respectively. Paternity analysis in CERVUS v.3.0.7 software (Kalinowski et al., 2007; Marshall et al., 1998) was used to verify the maternal pedigree corrections and paternity assignments. After filtering the 45,378 SNPs for MAF of 0.05, Hardy Weinberg Equilibrium (*α* = 0.001), and Linkage Disequilibrium (LD) of zero, 1,136 SNPs were retained and used in CERVUS. We run the “parent pair‐sexes known” analysis to verify the identified female and male parents using the ***G*** matrix. Two pedigree‐based additive relationship matrices (***A*** matrices) were compared: the original PX‐pedigree (***A***_PX_) and the reconstructed FS‐pedigree (***A***_FS_). Two dominance relationship matrices were compared: the corrected pedigree‐based dominance relationship matrix (***A***_d_ matrix) was computed using the R function “Amatrix”, and the realized dominance genomic relationship matrix (***G***_d_ matrix) was calculated following the method of Vitezica et al. (2013) using the R function “Gmatrix” from the R package “AGHmatrix” (Amadeu et al., 2016). The Pearson's correlation between all additive relationship matrices was estimated to verify their similarity (i.e., to look for outlier correlations). We also compared between the expected average relationship from FS‐ and PX‐ pedigrees, and the average of realized genomic relationship from the ***G*** matrix. All analyses were done in the R v.4.0.2 environment (R Core Team, 2020).

### 2.5 Genetics models and parameters

For each trait, we fitted five individual tree models (Isik et al., 2017): three pedigree‐based models (ABLUP‐PX, ABLUP‐FS‐A, and ABLUP‐FS‐AD) and two genomics‐based models (GBLUP‐A and GBLUP‐AD). We fitted all models in ASReml‐R v.4.1 (Butler et al., 2017; Butler, 2020) and we computed variance components, narrow‐sense heritability (${\hat{h}}^{2})$ and broad‐sense heritability $\left({\hat{H}}^{2}\right)$, type‐B additive genetic correlation (${\hat{r}}_{B}$), BVs, and Akaike Information Criterion (AIC) from each model.

We first fitted the following three mixed‐effect linear models (ABLUP‐PX, ABLUP‐FS‐A and GBLUP‐A) including the additive effect only:

(1) $$y=X\beta +Z_{1}r\left(s\right)+Z_{2}\mathrm{ib}\left(r\right)+Z_{3}a+Z_{4}\mathrm{sa}+e$$

where y is the vector of measured phenotypes; β are the vectors of the fixed effects including site means, heartwood width (mm), and total number of rings used as covariates for wood traits only; $r\left(s\right)$ is the vector of random replicate (block) nested within site effect; $\mathrm{ib}\left(r\right)$ is the vector of random incomplete block (set) nested within replicate (block) effect; a is the vector of random additive genetic effect (BVs) estimated from ***A***_PX_, ***A***_FS_, or ***G***_all_ matrix; sa is the vector of random interaction of site with additive genetic effects; and e is the vector of random heterogenous residuals. ***X*** and ***Zs*** are incidence matrices relating the phenotypes to the model terms. More detailed descriptions about all the random model terms and their variances distributions are available in Appendix S1.

Next, we extended the corrected pedigree and genomic additive models (ABLUP‐FS‐A and GBLUP‐A) to include the dominance effect and its interaction as follows:

(2) $$y=X\beta +Z_{1}r\left(s\right)+Z_{2}\mathrm{ib}\left(r\right)+Z_{3}a+Z_{4}\mathrm{sa}+Z_{5}d+Z_{6}\mathrm{sd}+e$$

where d is the vector of random dominance genetic effect estimated from the ***A***_d_ or ***G***_d_ matrix; and sd is the vector of random interaction of site with dominance genetic effects. The other terms are as defined in Equation (1). More detailed descriptions about the additional random model terms and their variances distributions are available in Appendix S1. The significance of each genetic variance component ($\sigma_{a}^{2}$, $\sigma_{\mathrm{sa}}^{2}$, $\sigma_{d}^{2}$ and $\sigma_{\mathrm{sd}}^{2}$) was tested in the five models using a likelihood ratio test (LRT) with one degree of freedom (*df*) between the full model in Equation (1) or (2), and a reduced model missing the tested single term only. We used the function “lrt” in the R package “asreml” for LRT (Butler, 2020). We estimated ${\hat{h}}^{2}$, ${\hat{H}}^{2}$, and ${\hat{r}}_{B}$ (magnitude of genotype by environment [G × E] interaction) for additive genetic effects, as described in Appendix S1. We also estimated Pearson's correlations between the estimated BVs from the five models for OTs. Moreover, we estimated the Pearson's and Spearman's correlations between FPT BVs from ABLUP‐PX and ABLUP‐FS‐A models, to check the effect of maternal pedigree errors on FPT BV ranking. Theoretical accuracy ($\hat{r}$), which is the square root of reliability (the correlation between true BV and estimated BV), is used to evaluate models with complete data sets and not from cross‐validation (Mrode, 2014). We estimated ($\hat{r}$) of FPTs, MPTs, and OTs BVs for the three additive models using the following formula:

(3) $$\hat{r}=\sqrt{1-\frac{{{\mathrm{SE}}_{i}}^{2}\;}{\left(\;1+F_{i}\right)\;{\hat{\sigma }}_{a}^{2}}}$$

where, SE_*i*_ is the BV standard error, *F*_*i*_ is the inbreeding coefficient for the *i*th individual and ${\hat{\sigma }}_{a}^{2}$ is the estimated additive genetic variance, and (1 + *F*_*i*_) is the diagonal of additive relationship matrix (***A***_PX_ for ABLUP‐PX, ***A***_FS_ for ABLUP‐FS‐A, and ***G***_all_ for GBLUP‐A). To make $\hat{r}$ comparable between the two pedigree models (ABLUP‐PX and ABLUP‐FS‐A) and the genomic model (GBLUP‐A), we fixed the values of $\sigma_{a}^{2}$ and $\sigma_{\mathrm{sa}}^{2}$ in Equation (1) in the two pedigree models to the values estimated from GBLUP‐A (El‐Kassaby et al., 2011; Lenz, Nadeau, Azaiez et al., 2020).

### 2.6 Correlation between traits

Phenotypic correlations were estimated as the Pearson's correlation between the adjusted phenotypes y* (the residual from a model similar to Equation (1), but without the two genetic terms $Z_{3}a+Z_{4}\mathrm{sa}$) of the eight traits. Instead of performing 28 bivariate models to estimate the genetic correlation, we ran eight multi‐variate GBLUP‐A models (four penta‐variate, one tri‐variate and three bivariate models) using CORGH variance structure for the additive and residual terms to get an estimate for the additive genetic correlation between the eight traits. The multivariate model is described in Appendix S1.

### 2.7 Comparison between ABLUP‐PX and GBLUP expected genetic gains

We compared the three additive genetic models (ABLUP‐PX, ABLUP‐FS‐A, and GBLUP‐A) estimated mean BVs, BVs accuracy, and the expected genetic gain % from selecting the top 5% trees for each trait individually (i.e., with a census size, *N* = 75 trees). These estimated gains are considered the maximum possible gain without any optimal selection strategy, as no restrictions were applied for genetic diversity. Then, the status number (*N*s) was used as a measure for the genetic diversity of the selected set (top 75 trees), and was estimated following Lindgren et al.'s formula Lindgren et al. (1997):

(4) $$Ns=\frac{1}{2\theta }$$

where *θ* is the group coancestry. Then, we used the GBLUP‐A as our standard, to estimate the corrected BVs, % gain, and overlapped % with the pedigree models.

### 2.8 Comparing different SNPs types

SNP effects for all 45,378 SNPs were annotated using the Variant Effect Predictor (McLaren et al., 2016). We used all the 45,378 SNPs as the main genotypic file, which was further divided into three additional genotype files: genic‐coding, genic‐non‐coding, and intergenic SNPs. To compare between the four groups of SNPs, we (1) estimated the genotype (−1: homozygotes for reference allele, 0: heterozygotes, 1: homozygotes for alternate allele) proportion; (2) compared the relationship estimates from the four ***G*** matrices (***G***_all_, ***G***_gen‐cod_, ***G***_gen‐no‐cod_, and ***G***_intergen_) versus the corrected ***A*** matrix (***A***_FS_); (3) calculated the Pearson's correlations between all five additive relationship matrices; ***A***_PX_, ***A***_FS_, ***G***_all_, ***G***_gen‐cod_, ***G***_gen‐no‐cod_, and ***G***_intergen_; and (4) ran three additional GBLUP‐A models to compare between the four genomic models using ${\hat{h}}^{2}$ and AIC.

## 3 RESULTS

### 3.1 Paternity assignment and pedigree verification

**FIGURE 2:**
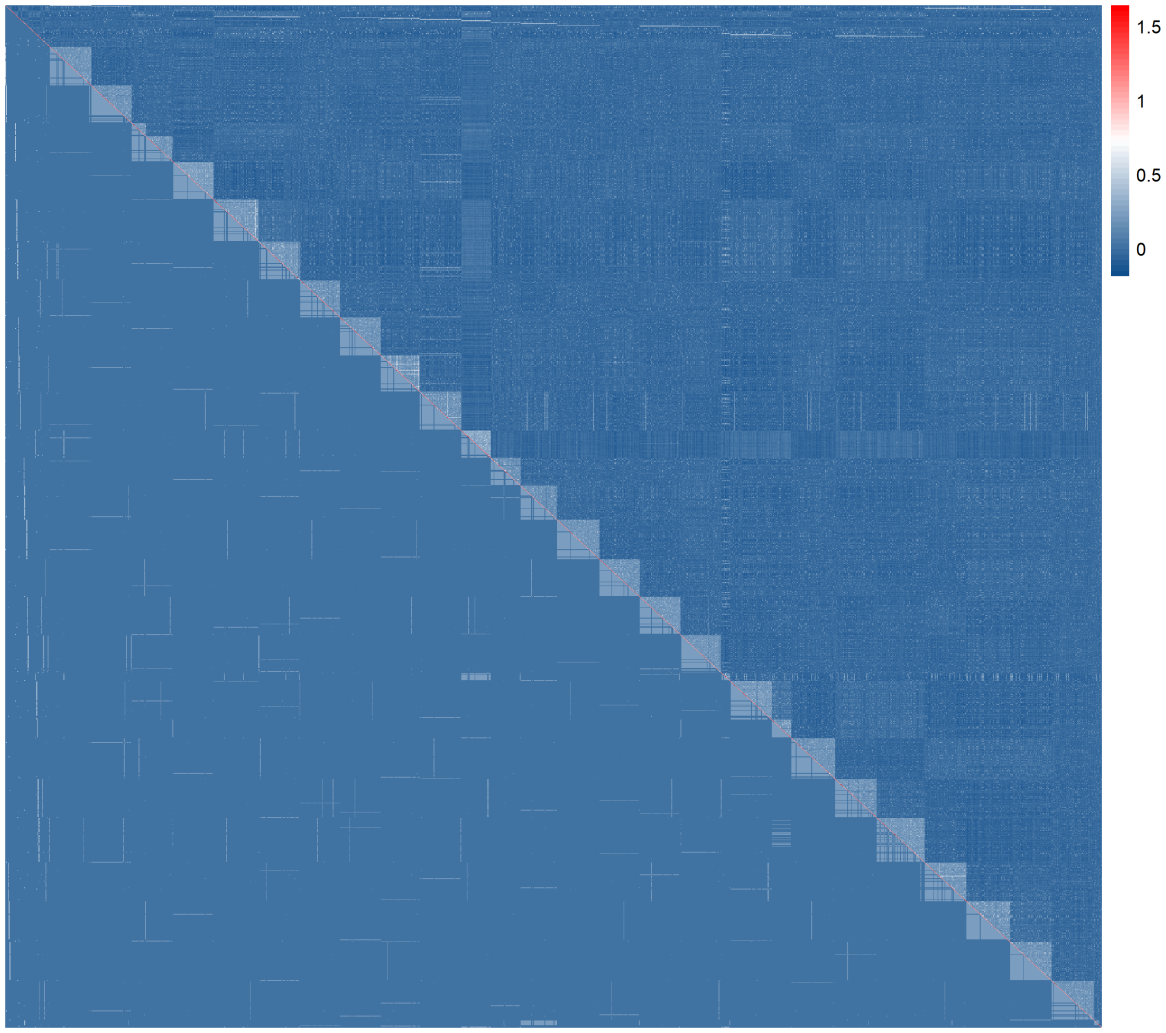
Heat‐map of pairwise PX‐pedigree (***A***_PX_ matrix in the lower diagonal) and genomic relationship matrix (***G*** matrix in the diagonal and upper diagonal) for parents and offspring. Parent relationships are on the top and offspring are ordered by corrected maternal families. We observe no relationship between parents (in the first 44 rows and columns) in both matrices. Parent‐offspring relationship can be seen in the first 44 columns (in the ***A***_PX_ matrix) and the first 44 rows (in the ***G*** matrix). The upper diagonal (***G*** matrix) shows ideal HS relationship within corrected maternal families, which is represented by the squared matrices on the diagonal, and scattered HS and FS relationships in the remaining upper off‐diagonals. The lower diagonal (***A***_PX_ matrix) shows pedigree errors in the form of a lot of unrelated individuals within the squared matrices on the diagonal (corrected maternal families), and incorrect HS‐relationship (in scattered lines) in the remaining lower off‐diagonals.

**FIGURE 3:**
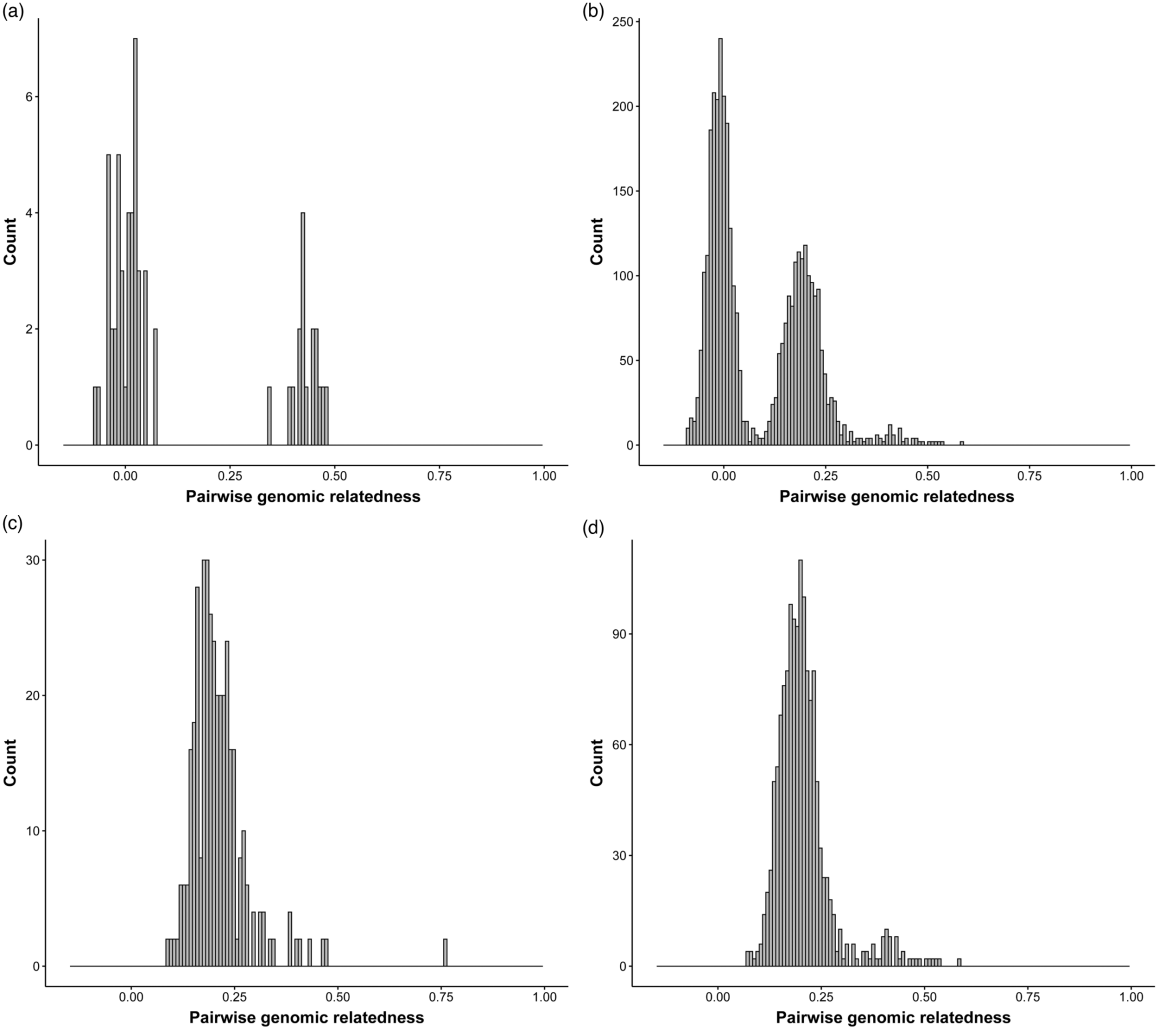
Histogram of pairwise genomic relationships for one out of the eight maternal families showing two possible genotypes. (a) Parent‐offspring relationship; showing two clusters, the peak at 0 relationship coefficient represents the offspring group not related to the genotyped parent, while the peak, around 0.4 relationship coefficient, represents the offspring group related to the genotyped parent. (b) Offspring‐offspring relationship within the same family; showing two clusters, the peak at 0 relationship coefficient represents the half‐sib offspring group not related to each other, while the peak around 0.2 relationship coefficient represent the HS offspring group related to each other. (c and d) Offspring‐offspring relationship of the two groups separately showing the disappearance of the peak at 0 relationship coefficient, and half‐sib relationship within each new corrected maternal family around 0.2 relationship coefficient.

**FIGURE 4:**
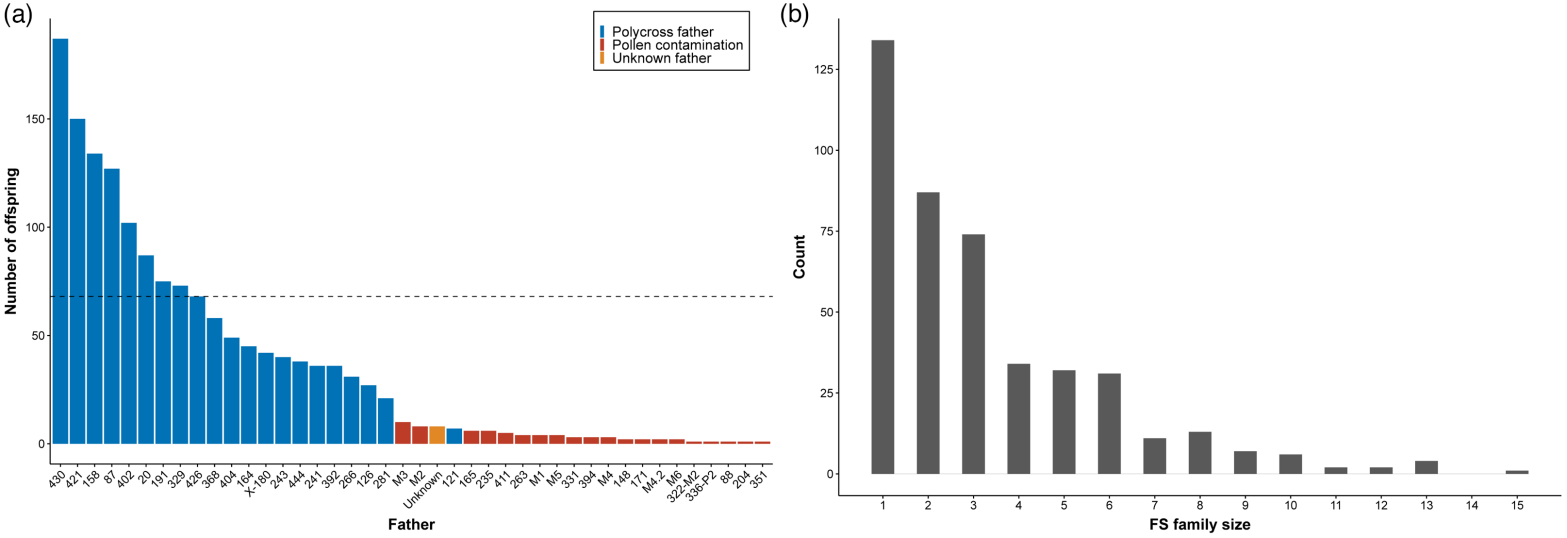
Unequal male contribution leads to unbalanced FS family sizes. (a) Histogram of unequal male contribution. Number of offspring per pollen donor ranges from 7 to 181. The dashed line represents the expected equal male contribution of 68 offspring per pollen donor. The blue bars represent the 1433 (out of 1510) trees assigned one of the 21 males used in the pollen mix. The red and yellow bars represent pollen contamination; yellow bar represents 8 trees who were not assigned to any male parent; red bars represent trees identified as selfs or assigned foreign males other than the 21 males in the pollen mix, which were identified from sib‐sib analysis or parent‐offspring relationship with other genotyped parents. (b) Histogram of full‐sib (FS) family size distribution showing small and unbalanced sizes ranged from 1 to 15 offspring per family, total of 438 FS families.

The ***G*** matrix (***G***_all_), representing the realized additive genomic relationship between all parents and OTs, is shown in Figure 2. All relationships were confirmed from parentage and sibship analyses, except one maternal (dead and not available for genotyping) and paternal family (mislabeled). The parent‐offspring and offspring‐offspring genomic relationships ranged from 0.17 to 0.59, and from 0.08 to 0.43, respectively (Table S2). Pedigree correction resulted in 28 individuals (1.85%) of the 1510 OTs not being assigned to any of the expected 26 FPTs. Paternity was successfully reconstructed for 1433 OTs (95%), while the remaining 77 OTs (5%) were identified as resulting from pollen contamination (Figure 4a). Two pairs in the OTs were identified as duplicates with a relatedness coefficient of 0.89 and 0.92, and were removed from the study, reducing the number of OTs to 1506. Unexpectedly, we identified eight (out of 26) FPTs with two possible genotypes. This error was detected by identifying two relationship groups within each of these maternal families (please see example in Figure 3). There are two relationship clusters in parent‐offspring relationship (Figure 3a), and in offspring‐offspring relationship (Figure 3b). We divided each of these families into two separate HS maternal families, which were confirmed from the offspring‐offspring relationship within each separated HS family, and the absence of any relationship at 0 relationship coefficient (Figure 3c,d). Accounting for this issue raised the estimated pedigree error on the female side from 12.45% to 31.26%. CERVUS fully agreed with all the ***G*** matrix maternal pedigree corrections and paternity assignments for all offspring with genotyped parents. As expected, it did not assign any parents for all offspring with one or both ungenotyped parents identified in the ***G*** matrix (28 OTs with no identified FPT and 77 OTs resulted from pollen contamination).

After excluding 77 OTs due to pollen contamination, paternity assignment revealed significant unequal reproductive success deviating from the expected equal male contribution (chi‐squared statistics [*χ* ^2^] = 654.91, *df* = 20, *p*‐value < 2.2e‐16). The number of OTs/MPT ranged from 7 to 187 (mean = 68, SD = 47, Figure 4a). A total of 1429 out of 1506 OTs (95% of OTs) were assigned to an expected FPT and MPT, resulting in 438 FS families, while the remaining OTs (5%) have at least one foreign parent (outside the expected 45 parents) or are selfed trees. Paternity assignment converted the 26 PX large families (i.e., families with 38–61 OTs/FPT, mean = 58) to 438 FS families (1–15 OTs/FS family, mean = 3.3, Figure 4b).

### 3.2 Genetics estimates

#### 3.2.1 Heritabilities

The additive genetic effect ($\sigma_{a}^{2}$) was significantly greater than zero in all of the five models for all eight traits (Table 1). Across the five models, foliar traits were the most heritable (${\hat{h}}^{2}$: 0.25–0.34) followed by wood traits (${\hat{h}}^{2}$: 0.12–0.29), while growth traits were the least heritable (${\hat{h}}^{2}$: 0.07–0.17) (Table 2). The largest differences in ${\hat{h}}^{2}$ and its SE was observed between ABLUP‐PX and the other models.

**TABLE 1:** Variance components^a^ (standard errors in parentheses), and their significance^b^ obtained from the five studied models

	HT	DBH	F.AT	F.TM	W.AT	W.TT	W.TL	W.TE
ABLUP‐PX‐A								
${\hat{\sigma }}_{a}^{2}$	2002 (903)***	64 (35)***	14,826,426 (5,558,702)***	30,657,455 (12,653,538)***	0.23 (0.08)***	0.15 (0.06)***	0.13 (0.05)***	0.14 (0.05)***
${\hat{\sigma }}_{\mathrm{sa}}^{2}$	640 (615)	14 (31)	4,015,174 (2,752,585)*	12,452,425 (7,787,821)*	0.00 (NA)	0.00 (NA)	0.00 (NA)	0.00 (NA)
${\hat{\sigma }}_{e}^{2}$	9057 (1127)	578 (57)	26,876,824 (5,393,815)	75,372,139 (13,250,043)	0.54 (0.08)	0.51 (0.06)	0.66 (0.07)	0.35 (0.05)
ABLUP‐FS‐A								
${\hat{\sigma }}_{a}^{2}$	1556 (566)***	51 (26)***	15,533,815 (4,147,436)***	37,118,801 (10,176,402)***	0.12 (0.04)***	0.09 (0.03)***	0.12 (0.04)***	0.09 (0.03)***
${\hat{\sigma }}_{\mathrm{sa}}^{2}$	705 (417)*	49 (25)**	1,689,946 (1,443,232)	4,366,040 (3,956,406)	0.04 (0.03)	0.03 (0.02)	0.00 (NA)	0.03 (0.02)*
${\hat{\sigma }}_{e}^{2}$	9341 (882)	550 (49)	29,077,111 (3,687,699)	78,932,462 (9,646,475)	0.62 (0.06)	0.55 (0.05)	0.68 (0.06)	0.38 (0.04)
GBLUP‐A								
${\hat{\sigma }}_{a}^{2}$	1468 (474)***	58 (26)***	11,737,942 (2,501,200)***	27,454,351 (6,346,220)***	0.14 (0.04)***	0.12 (0.03)***	0.11 (0.03)***	0.10 (0.03)***
${\hat{\sigma }}_{\mathrm{sa}}^{2}$	824 (430)**	56 (27)***	2,930,377 (1,783,734)*	7,717,227 (4,843,862)*	0.06 (0.03)*	0.05 (0.03)*	0.00 (NA)	0.04 (0.02)**
${\hat{\sigma }}_{e}^{2}$	8753 (887)	511 (51)	27,662,309 (3,389,231)	75,605,758 (9,098,886)	0.54 (0.06)	0.47 (0.05)	0.67 (0.06)	0.33 (0.04)
ABLUP‐FS‐AD								
${\hat{\sigma }}_{a}^{2}$	1452 (565)***	50 (26)***	15,533,579 (4,149,005)***	37,123,981 (10,167,817)***	0.11 (0.04)***	0.08 (0.03)***	0.11 (0.04)***	0.08 (0.03)***
${\hat{\sigma }}_{\mathrm{sa}}^{2}$	721 (428)*	47 (26)**	1,689,637 (1,443,162)	4,361,816 (4,045,090)	0.04 (0.03)	0.03 (0.02)	0.00 (NA)	0.03 (0.02)*
${\hat{\sigma }}_{d}^{2}$	1076 (1158)	0 (NA)	18 (NA)	51 (NA)	0.12 (0.07)*	0.11 (0.06)*	0.05 (0.07)	0.06 (0.04)*
${\hat{\sigma }}_{\mathrm{sd}}^{2}$	53 (1879)	34 (116)	36 (NA)	15 (NA)	0.00 (NA)	0.00 (NA)	0.00 (NA)	0.00 (NA)
${\hat{\sigma }}_{e}^{2}$	8274 (1701)	517 (100)	29,077,853 (3,688,188)	78,933,337 (18,454,588)	0.50 (0.09)	0.45 (0.07)	0.64 (0.08)	0.32 (0.05)
GBLUP‐AD								
${\hat{\sigma }}_{a}^{2}$	1158 (447)***	42 (25)***	11,737,609 (2,501,338)***	27,414,139 (6,351,165)***	0.14 (0.04)***	0.11 (0.03)***	0.10 (0.03)***	0.09 (0.03)***
${\hat{\sigma }}_{\mathrm{sa}}^{2}$	694 (429)*	56 (27)**	2,929,279 (1,783,558)*	7,720,452 (4,919,950)*	0.06 (0.03)*	0.06 (0.03)*	0.00 (NA)	0.04 (0.02)**
${\hat{\sigma }}_{d}^{2}$	1049 (853)**	81 (51)*	8 (NA)	22 (NA)	0.04 (0.05)	0.06 (0.04)	0.13 (0.06)*	0.04 (0.03)
${\hat{\sigma }}_{\mathrm{sd}}^{2}$	1685 (1177)*	17 (65)	4 (NA)	56 (NA)	0.00 (NA)	0.00 (NA)	0.00 (NA)	0.00 (NA)
${\hat{\sigma }}_{e}^{2}$	6044 (1249)	417 (74)	27,663,310 (3,389,321)	75,623,294 (13,188,818)	0.49 (0.07)	0.41 (0.06)	0.54 (0.09)	0.30 (0.05)

*Note*: The models tested are: “ABLUP‐PX‐A” is the PX pedigree‐based model (using the ***A*** matrix estimated from the PX pedigree with known mothers and unknown fathers); “ABLUP‐FS‐A” is the FS pedigree‐based model (using the ***A*** matrix estimated from the corrected pedigree with known mothers and fathers); “GBLUP‐A” is the genomic selection model (using the realized additive genomic relationship matrix ***G***, estimated from SNPs); “ABLUP‐FS‐AD” is the FS pedigree‐based model (using the average additive and dominance relationship matrices, ***A*** and ***A***_d_ matrices, estimated from the corrected FS pedigree); “GBLUP‐AD” is the genomic selection model (using the realized additive and dominance genomic relationship matrices, ***G*** and ***G***_d_ matrices, estimated from SNPs).

^a^ ${\hat{\sigma }}_{a}^{2}$ = additive variance; ${\hat{\sigma }}_{\mathrm{sa}}^{2}$ = site‐by‐additive interaction variance; ${\hat{\sigma }}_{d}^{2}$ = dominance variance; ${\hat{\sigma }}_{\mathrm{sd}}^{2}$ = site‐by‐dominance interaction variance; ${\hat{\sigma }}_{e}^{2}$ = the average of the heterogenous residual variances of the three sites.

^b^ Significance levels for testing genetic variance components using likelihood ratio test: *p < 0.05; **p < 0.01; ***p < 0.001.

**TABLE 2:** Estimates of individual narrow‐sense heritability (${\hat{h}}^{2}$, SE) broad‐sense heritability (${\hat{H}}_{\mathrm{ind}}^{2}$, SE), type‐B genetic correlation (${\hat{r}}_{B}$, SE) and Akaike Information Criterion (AIC) for all models and tested traits

Trait	Model		ABLUP		GBLUP	
	Parameters	‐PX	‐FS‐A	‐FS‐AD	‐A	‐AD
HT	${\hat{h}}^{2}\;$	0.17 (0.07)	0.13 (0.05)	0.13 (0.05)	0.13 (0.04)	0.11 (0.04)
	${\hat{H}}^{2}$	–	–	0.22 (0.10)	–	0.21 (0.08)
	${\hat{r}}_{B\;}$	0.76 (0.21)	0.69 (0.17)	0.67 (0.18)	0.64 (0.16)	0.63 (0.20)
	AIC	15,822	15,796	15,798	15,785	**15,777**
DBH	${\hat{h}}^{2}\;$	0.10 (0.05)	0.08 (0.04)	0.08 (0.04)	0.09 (0.04)	0.07 (0.04)
	${\hat{H}}^{2}$	–	–	0.08 (0.10)	–	0.20 (0.08)
	${\hat{r}}_{B\;}$	0.82 (0.36)	0.51 (0.21)	0.52 (0.22)	0.51 (0.19)	0.43 (0.22)
	AIC	11,465	11,444	11,446	11,436	**11,435**
F.AT	${\hat{h}}^{2}\;$	0.32 (0.11)	0.34 (0.08)	0.34 (0.08)	0.28 (0.05)	0.28 (0.05)
	${\hat{H}}^{2}$			0.34 (0.08)		0.28 (0.05)
	${\hat{r}}_{B\;\;}$	0.79 (0.14)	0.9 (0.08)	0.9 (0.08)	0.8 (0.11)	0.8 (0.11)
	AIC	28,105	**28,034**	28,034	28,037^a^	28,037
F.TM	${\hat{h}}^{2}\;$	0.26 (0.10)	0.31 (0.07)	0.31 (0.07)	0.25 (0.05)	0.25 (0.05)
	${\hat{H}}^{2}$			0.31 (0.07)		0.25 (0.05)
	${\hat{r}}_{B\;}$	0.71 (0.17)	0.89 (0.09)	0.89 (0.09)	0.78 (0.12)	0.78 (0.13)
	AIC	29,641	**29,575**	29,575	29,578^a^	29,578
W.AT	${\hat{h}}^{2}\;$	0.29 (0.10)	0.15 (0.05)	0.14 (0.05)	0.19 (0.05)	0.19 (0.05)
	${\hat{H}}^{2}$			0.30 (0.10)		0.25 (0.08)
	${\hat{r}}_{B\;}$	1 (0)	0.77 (0.17)	0.75 (0.17)	0.71 (0.15)	0.71 (0.15)
	AIC	1191	1200^b^	1199	**1186**	1187
W.TT	${\hat{h}}^{2}\;$	0.23 (0.08)	0.13 (0.05)	0.12 (0.05)	0.18 (0.05)	0.18 (0.05)
	${\hat{H}}^{2}$	–	–	0.28 (0.09)	–	0.27 (0.08)
	${\hat{r}}_{B\;}$	1 (NA)	0.76 (018)	0.74 (0.19)	0.68 (0.15)	0.67 (0.15)
	AIC	1016	1011	1008	**994**	994
W.TL	${\hat{h}}^{2}\;$	0.17 (0.07)	0.15 (0.04)	0.14 (0.04)	0.14 (0.04)	0.13 (0.04)
	${\hat{H}}^{2}$			0.20 (0.09)		0.29 (0.08)
	${\hat{r}}_{B\;}$	1 (0)	1 (0)	1 (0)	1 (0)	1 (0.23)
	AIC	1257	1244	1246	1240	**1237**
W.TE	${\hat{h}}^{2}\;$	0.28 (0.09)	0.17 (0.05)	0.16 (0.05)	0.20 (0.05)	0.19 (0.05)
	${\hat{H}}^{2}$			0.29 (0.10)		0.28 (0.08)
	${\hat{r}}_{B\;}$	1 (0)	0.77 (0.15)	0.76 (0.15)	0.69 (0.14)	0.68 (0.14)
	AIC	567	554	554	**539**	540

*Note*: Bold AIC: The smallest AIC (the best model in term of goodness of fit).

Abbreviations: DBH, diameter at breast height; HT, height; F.AT, foliar α‐thujone; F.TM, foliar total monoterpenes (the sum of all monoterpenes); W.AT, wood α‐thujaplicin; W.TE, wood total extractives (the sum of W.TT and W.TL); W.TL, wood total lignans (the sum of plicatic acid and plicatin); W.TT, wood total thujaplicins (the sum of α‐, β‐, γ‐thujaplicin, and β‐thujaplicinol).

^a^ This unexpected three units increase in AIC for foliar traits in GBLUP‐A compared to ABLUP‐FS‐A, could be justified by ${\hat{h}}^{2}$ overestimation (by 0.06 which is the biggest difference across all traits), which may mislead to a better goodness of fit for ABLUP‐FS‐A.

^b^ This unexpected nine units increase in AIC for W.AT in ABLUP‐FS‐A compared to ABLUP‐PX could be explained by ${\hat{h}}^{2}$ overestimation (by 0.14, which is the biggest difference across all traits), resulting in an increase in the total variance explained by the model (reducing residual variance, Table S2) and falsely suggesting a better goodness of fit for ABLUP‐PX.

For growth and wood traits, ABLUP using the FS‐pedigree (ABLUP‐FS‐A) gave a smaller ${\hat{h}}_{\mathrm{ind}}^{2}$ than using PX‐pedigree (ABLUP‐PX). An opposite trend was observed for foliar traits. Regardless of whether or not ABLUP‐FS‐A increased or decreased ${\hat{h}}^{2}$ compared to ABLUP‐PX, the ${\hat{h}}^{2}$ standard error (SE) was lower by 0.01 (DBH) to 0.05 (W.AT), reflecting more accurate estimates. The better performance of the ABLUP‐FS‐A model, relative to the ABLUP‐PX, is also reflected in the AIC estimates, where ABLUP‐FS‐A estimates were lower than ABLUP‐PX. AIC decreased by 5 (W.TT) to 71 (F.AT) units for all traits except W.AT (Table 2).

GBLUP‐A and ABLUP‐FS‐A yielded the same ${\hat{h}}^{2}$ for HT, but SE in GBLUP‐A was one unit smaller, while for DBH the ${\hat{h}}^{2}$ was one unit larger in GBLUP‐A with the same SE. Foliar traits, however, showed a decrease in ${\hat{h}}^{2}$ (by 0.06) and SE (by 0.02–0.03) in GBLUP‐A. Surprisingly, wood traits (W.AT, W.TT, and W.TE) showed higher ${\hat{h}}^{2}$ (by 0.03–0.05) with the same SE in the GBLUP‐A, while W.TL showed a one unit decrease in ${\hat{h}}^{2}$ with the same SE. AIC for GBLUP‐A was smaller than ABLUP‐FS‐A (by 4–17 units) for all traits (except foliar monoterpenes trait), supporting the better performance of GBLUP‐A.

#### 3.2.2 Dominance significance (GBLUP‐A vs. GBLUP‐AD)

LRT showed that by using pedigree (ABLUP‐FS‐AD), the dominance genetic effect ($\sigma_{d}^{2}$) was significant for W.AT, W.TT, and W.TE. Meanwhile, the genomic approach (GBLUP‐AD) yielded a significant dominance genetic effect ($\sigma_{d}^{2}$) for HT, DBH, and W.TL (Tables 1 and 2). The significance of dominance effects was supported by smaller AIC for these traits. Given the unbalanced and small FS family structure, GBLUP‐AD is likely more accurate than ABLUP‐AD in estimating dominance effects. Only HT showed significant dominance interaction with environment ($\sigma_{\mathrm{sd}}^{2}$) in the GBLUP‐AD model. For HT, DBH, and W.TL, ${\hat{H}}^{2}$ and its SE were around double the ${\hat{h}}^{2}$ and its SE. A very small decrease was observed in the ${\hat{h}}^{2}$ (by 0.01–0.02) in the GBLUP‐AD model compared to GBLUP‐A.

#### 3.2.3 Type‐B additive genetic correlation and G × E interaction

Compared to the other four models, the ABLUP‐PX overestimated ${\hat{r}}_{B}$ for HT, DBH, W.AT, W.TT and W.TE (${\hat{r}}_{B}$ = 0.76, 0.82, 1, 1, and 1, respectively, Table 2), and differed with the GBLUP models in the significance of $\sigma_{\mathrm{sa}}^{2}$, leading to the erroneous conclusion that there is no G × E for these traits (Tables 1 and 2). However, W.TL showed a complete absence of G × E with ${\hat{r}}_{B}$ = 1 and ${\hat{\sigma }}_{\mathrm{sa}}^{2}=0$ across the five models, resulting in the highest tree rank stability across the three sites among all traits (Tables 1, and 2). Ranking traits for G × E, using the GBLUP‐A model, growth traits showed the highest G × E (i.e., lowest ${\hat{r}}_{B}$), followed by wood traits, while foliar traits were the lowest. ABLUP‐FS‐A, ABLUP‐FS‐AD and GBLUP‐AD gave the same G × E rank as GBLUP‐A (Table 2). All seven traits showed significant ${\hat{\sigma }}_{\mathrm{sa}}^{2}$ in the GBLUP models, while only HT, DBH, and W.TE showed significant ${\hat{\sigma }}_{\mathrm{sa}}^{2}$ in the ABLUP‐FS models (Table 1).

### 3.3 Model similarity and accuracy of breeding value estimates

ABLUP‐FS‐A and GBLUP‐A BVs were highly correlated and ranged from 0.91 (W.TT) to 0.96 (F.AT and F.TM) (Table S3); however, as expected the ABLUP‐PX had a smaller correlation with GBLUP‐A, ranging from 0.71 (W.TL) to 0.82 (F.AT and F.TM). A very similar correlation was observed with ABLUP‐FS‐A (Table S3). It is noted that foliar traits with the highest ${\hat{h}}^{2}$ gave the highest correlation, reflecting their lesser effect by the missing paternal information and pedigree errors than the lower heritability traits. The same pattern of the highest correlations for foliar traits was also observed in comparing FPT BVs across ABLUP‐PX and ABLUP‐FS‐A, indicating a lesser effect on FPT BVs ranking due to maternal pedigree errors (Table S8). Surprisingly, for all traits (whether the dominance effect was significant or not) the correlation between BVs from additive and additive‐dominance models in both pedigree and genomic models was equal to 1 (Table S3). Considering this, we estimated BV theoretical accuracies and expected genetic gain for the three additive models only (ABLUP‐PX, ABLUP‐FS‐A, GBLUP‐A).

Pedigree reconstruction and genomic analysis provided the opportunity to estimate MPTs BVs and their accuracies. Mean $\hat{r}$ for male BVs was very similar to that of females (Table 3 and Figure S5), the main difference being that they showed greater variation in accuracy (Table S4 and Figure S5). FPTs and MPTs' BV $\hat{r}$ obtained from GBLUP‐A was smaller than the pedigree‐based models (Table 3 and Table S4). For five traits, an increase of 0.01 was observed in FPTs' BV $\hat{r}$ when using the FS‐pedigree compared to PX‐pedigree (Table 3 and Table S4). In contrast, OTs BV $\hat{r}$ showed a large increase of 0.09–0.11 (16%–27%) in accuracy when using the FS‐pedigree compared to PX‐pedigree (Table 3). GBLUP‐A gave very similar BVs accuracy estimates relative to ABLUP‐FS‐A for OTs (Table 3). In GBLUP‐A, BV accuracy increased by 17% (for F.AT and W.AT) to 22% (DBH), when compared to ABLUP‐PX (Table S5). Across the three models, parents showed higher $\hat{r}$ for BVs (from 0.56 to 0.85) than OTs (from 0.41 to 0.67), as expected (Table 3 and Figure S5).

**TABLE 3:** Estimates of theoretical accuracy ($\hat{r}$) of parents' and offspring's breeding values for selected models and all tested traits

Model	ABLUP‐PX		ABLUP‐FS‐A			GBLUP‐A		
Trait^a^	Parents	Offspring	Parents		Offspring	Parents		Offspring
	Females		Males	Females		Males	Females	
HT	0.74	0.47	0.73	0.74	0.58	0.63	0.63	0.56
DBH	0.67	0.41	0.66	0.67	0.52	0.56	0.56	0.50
F.AT	0.84	0.58	0.83	0.85	0.67	0.71	0.72	0.67
F.TM	0.82	0.55	0.81	0.83	0.65	0.70	0.70	0.65
W.AT	0.79	0.53	0.78	0.80	0.62	0.67	0.68	0.62
W.TT	0.78	0.52	0.77	0.79	0.61	0.66	0.67	0.61
W.TL	0.8	0.51	0.78	0.80	0.62	0.67	0.68	0.60
W.TE	0.79	0.53	0.78	0.80	0.62	0.67	0.68	0.62

*Note*: The number of female parents = 25 (26 – 1 non‐genotyped parent), number of male parents = 20 (21 – 1 non‐genotyped parent), number of offspring = 1506 (representing 26 PX families and 438 FS families after pedigree reconstruction), and the average number of offspring per family ≈58/maternal HS family, 68/paternal HS family, and 3.3/FS family.

^a^ See Table 1 for traits description.

### 3.4 Correlation between traits

Large significant positive phenotypic and genetic correlations (0.41–1) were observed within each trait category (i.e., growth, foliar and wood traits) (Table 4). For foliar traits (F.AT and F.TM), the phenotypic and genetic correlations were similar and equal at 0.97. As expected, the phenotypic correlations are lower than the genetic correlations for all traits. W.TL had the lowest phenotypic and genetic correlations with the other three wood traits (0.41–0.74), while the correlations among the other three traits (i.e., W.TT, W.AT and W.TE) were higher (0.81–1). The only significant moderate correlation observed between the three traits categories was the genetic correlation observed between W.TL, and growth traits (0.47, 0.43, for HT and DBH, respectively). The remaining correlations were small and mostly non‐significant (Table 4).

**TABLE 4:** Phenotypic (above diagonal) and genetic correlation (below diagonal) between tested traits

Trait^a^	HT	DBH	F.AT	F.TM	W.AT	W.TT	W.TL	W.TE
HT		**0.81**	0.05	0.04	−0.04	−*0.09*	−0.05	−*0.09*
DBH	**0.78**		*0.10*	*0.10*	−*0.08*	−*0.14*	−*0.13*	−*0.16*
F.AT	0.03	0.23		**0.97**	−0.02	−0.04	−*0.05*	−0.05
F.TM	0.02	0.21	**0.97**		0.00	−0.02	−*0.06*	−0.03
W.AT	0.16	0.03	0.14	0.16		**0.85**	**0.41**	**0.81**
W.TT	0.09	0.00	0.13	0.14	**0.82**		**0.66**	**0.97**
W.TL	**0.47**	**0.43**	−0.02	−0.01	**0.47**	**0.59**		**0.74**
W.TE	0.12	0.03	0.11	0.12	**0.83**	1.00	0.67	

*Note*: Significance of both correlations was assessed differently. For phenotypic correlation, we used cor.test function in *R* for the correlation between the adjusted phenotypes to estimate *p*‐value. We used an *α*‐level of 0.05 to determine significance. Bold type reflects strong significant correlation, while italics reflect small significant correlations. For genetic correlation estimated from multivariate GBLUP‐A models using CORGH structure, we identified significance as having a correlation estimate at least double the SE. Bold type reflects significant correlation. Correlation cut‐offs: small, <0.4; medium, 0.4–0.7; and strong, >0.7.

^a^ See Table 1 for traits description.

### 3.5 Comparison between ABLUP‐PX and GBLUP‐A expected genetic gains

GBLUP‐A showed the highest expected genetic gain for all traits and varied from 6.01% (HT) to 55% (W.TL) (Table 5). Surprisingly W.TL resulted in the highest expected genetic gain, followed by foliar traits with expected genetic gain, 25.5% and 22%, for F.AT and F.TM, respectively (Table 5). HT, DBH, W.AT, W.TT, and W.TE had expected gains ranging from 6.01% (HT) to 11% (W.TE) (Table 5).

**TABLE 5:** Comparison of expected genetic gains and corrected expected genetic gain for the selection of top 5% trees (census number = 75) using selected models for all tested traits

Trait^a^	Model	*N*s^b^ (FS‐ped)	Theoretical accuracy	Mean BV^c^	Gain (%)^d^	Corrected mean BV^e^	Corrected gain (%)	GBLUP intersection (%)^f^
HT	ABLUP‐PX	17.3 (PX‐ped) 13.4 (FS‐ped)	0.48	44.1	5.7	34.7	4.49	42.7
	ABLUP‐FS‐A	5.8	0.6	46.9	6.06	41.3	5.34	62.7
	GBLUP‐A	7.9	0.57	46.5	**6.01**	–	–	–
DBH	ABLUP‐PX	14.5 (PX‐ped) 12.9 (FS‐ped)	0.41	6.8	6.48	5.95	5.66	52
	ABLUP‐FS‐A	7.8	0.53	7.19	6.85	7.18	6.84	65.3
	GBLUP‐A	10.3	0.5	7.81	**7.44**	–	–	–
F.AT	ABLUP‐PX	22.2 (PX‐ped) 15.7 (FS‐ped)	0.58	5422	23.3	5299	22.8	66.7
	ABLUP‐FS‐A	11.8	0.67	6092	26.2	5615	24.2	73.3
	GBLUP‐A	11.3	0.68	5916	**25.5**	–	–	–
F.TM	ABLUP‐PX	21.8 (PX‐ped) 15.1 (FS‐ped)	0.55	7008	18	7561	19	64
	ABLUP‐FS‐A	11.2	0.65	9274	23	8297	21	76
	GBLUP‐A	11.2	0.65	8706	**22**	–	–	–
W.AT	ABLUP‐PX	14.2 (PX‐ped) 12.1 (FS‐ped)	0.54	0.49	8.6	0.33	5.7	45
	ABLUP‐FS‐A	7.4	0.64	0.38	6.7	0.4	6.9	61
	GBLUP‐A	10.8	0.63	0.44	**7.7**	–	–	–
W.TT	ABLUP‐PX	14.8 (PX‐ped) 12.1 (FS‐ped)	0.52	0.39	5.85	0.31	4.70	44
	ABLUP‐FS‐A	7.4	0.63	0.36	5.34	0.38	5.63	60
	GBLUP‐A	10.9	0.62	0.41	**6.15**	–	–	–
W.TL	ABLUP‐PX	13.8 (PX‐ped) 13.7 (FS‐ped)	0.51	0.39	49	0.33	41	48
	ABLUP‐FS‐A	8.1	0.63	0.44	54	0.42	52	71
	GBLUP‐A	9.4	0.61	0.44	**55**	–	–	–
W.TE	ABLUP‐PX	19.3 (PX‐ped) 14.3 (FS‐ped)	0.53	0.4	12	0.31	9.1	48
	ABLUP‐FS‐A	8.1	0.64	0.38	11	0.37	10.7	68
	GBLUP‐A	9.3	0.63	0.39	**11**	–	–	–

*Note*: Bold gain (%): GBLUP‐A genetic gain.

^a^ See Table 1 for traits description.

^b^ *N*s: Status number of the 75 selected trees calculated from Equation (4) using the corrected pedigree and the original pedigree (only for ABLUP‐PX).

^c^ Mean BV: The BV mean of the 75 selected trees.

^d^ Gains (%): Gains are expressed as the percentage of the selected 75 trees' mean BV relative to the population phenotypic mean.

^e^ Corrected mean BV: The BV mean of the same selected 75 trees but from GBLUP‐A model, which was used to estimate the corrected gain (%).

^f^ GBLUP overlap (%): The percentage of the overlapped trees between the selected 75 trees from ABLUP‐PX/FS‐A and GBLUP‐A.

Given that GBLUP‐A results in the most reliable genetic estimates, it was used as a reference to gauge overestimation of expected genetic gain estimates from ABLUP‐PX. First, we used the GBLUP‐A model to extract the corrected BVs for the selected 75 OT from the non‐reliable ABLUP‐PX model. Then, we estimated the corrected mean BV which was used to estimate the corrected gain (%) from the ABLUP‐PX model. Finally, we compared the original gain % estimate from the ABLUP‐PX to the corrected gain % to estimate the gain overestimation % from the ABLUP‐PX model. We followed the same corrected estimates procedure from the ABLUP‐FS model (please see Table 5 and Table S5 for detailed calculations). For all traits, the GBLUP‐A gains % was higher than the corrected gain % from the ABLUP‐PX and ABLUP‐FS models (Table 5). The *N*s estimate from the PX pedigree was compared to the corrected *N*s (estimated from the FS pedigree) to estimate *N*s overestimation % from the ABLUP‐PX model (Table S5). The % gain that was overestimated from ABLUP‐PX varied from −5% (for F.TM) to 51% (W.AT) (Table S5). After excluding foliar traits' trend of −5% gain overestimation but the highest *N*s overestimation, the lowest edge is 14% (DBH). The % of *N*s overestimation from the ABLUP‐PX model ranged from 1% (W.TL) to 44% (F.TM) (Table S5). GBLUP‐A greatly improved the expected genetic gain over the PX model by 12% (F.AT) to 35% (W.AT) and to a smaller extent over the ABLUP‐FS‐A model, by 3% (W.TE) to 13% (HT). However, the corrected estimates of *N*s from the ABLUP‐PX‐A, a proxy for genetic diversity estimates, is higher than GBLUP‐A and ABLUP‐FS‐A (the smallest estimate for *N*s) (Table S5).

### 3.6 Comparing between different SNPs types and number of SNPs

42,031 out of the 45,378 SNPs were annotated and separated according to their predicted variant effects and locations into genic‐coding (14,767), genic‐non‐coding (14,406), and intergenic (12,858). Intergenic SNPs showed the largest disparity in genotype proportions (% ranges: −1 [59–66]; 0 [23–27]; and 1 [12–14], Table S6), with the highest proportion of homozygotes for the reference allele (66%) and the lowest for heterozygotes (23%). ***G***_gen‐no‐cod_ gave the highest relatedness (Table S7). ***G***_intergen_ gave the smallest correlation with the others ***G*** matrices (0.92–0.93 vs. 0.97, Table 6). GBLUP‐A_intergen_ gave the highest ${\hat{h}}^{2}$, while GBLUP‐A_gen‐no‐cod_ gave the lowest for HT and W.AT (Table 7). However, F.AT showed the opposite trend. By using all SNPs (GBLUP‐A), ${\hat{h}}^{2}$ were smaller by 0.01 for HT, DBH and W.AT, and higher by 0.02 for F.AT. We used traits measured from one variable only to be more accurate in this comparison. For growth, we selected HT for this comparison, due to its higher ${\hat{h}}^{2}$ and smaller G × E. Considering the overlapping standard errors of ${\hat{h}}^{2}$ and that the differences between the other estimates in this comparison are small, it is difficult to conclude that these different genetic estimates are biologically significant.

**TABLE 6:** Correlation between all additive relationship matrices: pedigrees (***A*** matrix for PX and FS pedigrees) and genomics (***G*** matrix for all, genic‐coding, genic‐no‐coding and intergenic SNPs)

Matrices	***A***_PX_	***A***_FS_	***G***_all_	***G***_gen‐cod_	***G***_gen‐no‐cod_	***G***_intergen_
***A***_PX_						
***A***_FS_	0.54					
***G***_all_	0.54	0.9				
***G***_gen‐cod_	0.53	0.89	0.99			
***G***_gen‐no‐cod_	0.53	0.89	0.99	0.97		
***G***_intergen_	0.53	0.87	0.96	0.93	0.92	

**TABLE 7:** Comparison between GBLUP‐A models for different ***G*** matrices (all, genic‐coding, genic‐no‐coding and intergenic SNPs) using individual narrow‐sense heritability (${\hat{h}}_{\mathrm{ind}}^{2}$, SE), and Akaike Information Criterion (AIC) for all traits

Trait^a^	Parameters	‐A	‐A_gen‐cod_	‐A_gen‐no‐cod_	‐A_intergen_
HT	${\hat{h}}^{2}\;$	0.13 (0.04)	0.12 (0.04)	0.1 (0.03)	0.14 (0.04)
	AIC	15,785	15,789	15,792	**15,782**
DBH	${\hat{h}}^{2}\;$	0.09 (0.04)	0.09 (0.04)	0.07 (0.03)	0.09 (0.04)
	AIC	**11,436**	11,437	11,439	11,438
F.AT	${\hat{h}}^{2}\;$	0.28 (0.05)	0.24 (0.05)	0.26 (0.05)	0.22 (0.05)
	AIC	28,037	28,047	**28,036**	28,046
F.TM	${\hat{h}}^{2}\;$	0.25 (0.05)	0.21 (0.05)	0.22 (0.05)	0.22 (0.05)
	AIC	**29,578**	29,587	29,578	29,584
W.AT	${\hat{h}}^{2}\;$	0.19 (0.05)	0.15 (0.04)	0.14 (0.04)	0.19 (0.05)
	AIC	**1186**	1194	1191	1187
W.TT	${\hat{h}}^{2}\;$	0.18 (0.05)	0.17 (0.05)	0.13 (0.04)	0.18 (0.05)
	AIC	994	**993**	1000	997
W.TL	${\hat{h}}^{2}\;$	0.14 (0.04)	0.13 (0.04)	0.13 (0.04)	0.12 (0.03)
	AIC	**1240**	1240	1240	1248
W.TE	${\hat{h}}^{2}\;$	0.2 (0.05)	0.18 (0.05)	0.16 (0.04)	0.18 (0.05)
	AIC	**539**	540	544	545

*Note*: Bold AIC: The smallest AIC (the best model in term of goodness of fit).

^a^ See Table 1 for traits description.

## 4 DISCUSSION

Despite the operational advantages of the PX mating design in forest tree breeding, there could be substantial biases in genetic variance estimates, gain predictions and genetic diversity estimates. However, the outperformance of the reconstructed pedigree (ABLUP‐FS‐A) and genomic (GBLUP) models over the traditional PX (ABLUP‐PX) model in terms of heritabilities, G × E estimates, model fit, BV accuracy, and expected genetic gain and diversity, increases the applicability of PX mating design particularly for forward selections. Moreover, it allows for screening more parents (PX pollen donors) and increasing selection intensity for backward selection. GBLUP has the additional advantage of capturing the Mendelian Sampling Term (MST) by using the actual proportion of shared genome between individuals to estimate relationships. It also eliminates the need for paternity assignment and pedigree maternal correction. We also found that traits with lower ${\hat{h}}^{2}$ (growth and wood traits) are more biased by missing parental information and pedigree errors as demonstrated by lower correlation between ABLUP‐PX and ABLUP‐FS/GBLUP‐A (Tables S3 and S8). Considering this, these traits would benefit more from molecular breeding than the more heritable foliar traits, resulting in higher improvement in BV $\hat{r}$ and expected genetic gain.

### 4.1 Pedigree reconstruction and biased genetic estimates from PX

The use of the normality properties of the realized relationship coefficient in the ***G*** matrix to correct pedigree errors was proposed by Munoz et al. (2014). In our study, we also used this approach for paternity assignment. Our results showed significant unequal male contribution, which ranged from 7 to 187 OTs/MPT. This large range in male contributions with PX breeding schemes has been observed in previous studies (Doerksen & Herbinger, 2008; EI‐Kassaby & Ritland, 1992; Lenz, Nadeau, Azaiez et al., 2020; Moriguchi et al., 2009; Vidal et al., 2015, 2017; Wheeler et al., 2006). The relatively small number of males (21) compared to the 111 females in the original trial could be a major reason for this unbalance. For instance, as smaller deviations from average male reproductive contribution were detected in maritime pine when using a much larger pollen mix (43 and 47 males) for 49 females in two populations (Vidal et al., 2015). Total maternal pedigree error of 31.26% is relatively large. However, when the error due to the two possible genotypes for eight out of the 26 FPTs was eliminated, it was reduced to only 12.45%, which is comparable to other studies reporting an average of 10% pedigree error (Doerksen & Herbinger, 2008, 2010; Munoz et al., 2014). This unexpected maternal error is most likely due to human error, which is expected during operational tree breeding (Godbout et al., 2017). There are two possible hypotheses which could explain this error. The first, is that the breeding was done on two clones and that one of them was mislabeled. The second, is that the two genotypes exist on the same clone, as the rootstalk can also produce cones in addition to the original tree if errors in pruning back rootstock were made.

Several studies reported the overestimation of ${\hat{h}}^{2}$ in PX and OP populations after pedigree reconstruction (Doerksen & Herbinger, 2010; Lenz, Nadeau, Azaiez et al., 2020; Vidal et al., 2015). A study by Klápště et al. (2017) in an OP advanced Eucalyptus breeding population found a mixed pattern of increasing and decreasing ${\hat{h}}^{2}$. Similarly, we found ${\hat{h}}^{2}$ to be either under‐ (foliar traits) or over‐ (growth and wood traits) estimated. Moreover, G × E was underestimated in ABLUP‐PX was in agreement with another study using PX in white spruce (Lenz, Nadeau, Azaiez et al., 2020). The PX‐pedigree underestimates all relationship classes (i.e., 0.00, 0.07, 0.19, 0.54, and 1, while expected average values are 0 for unrelated individuals, 0.25 for HS, 0.5 for FS and parent‐offspring, 1 for relationship matrix's diagonal of outbreed individual, and 1.5 for relationship matrix's diagonal of self individual, respectively) in the FS pedigree (Table S7). This large underestimation of all relationship classes, due to the inability to identify paternal HS, FS, and selfed individuals, and the large % of pedigree error, may explain the observed low correlation between ***A***_PX_ and ***A***_FS_ and all four ***G*** matrices (ranged from 0.53–0.54). Thus, ABLUP‐PX model results in significantly biased genetic estimates which translate to biased genetic gain (up to 51%) and *N*s (up to 44%) estimates.

### 4.2 Genetic estimates from GBLUP

#### 4.2.1 Heritability (ABLUP‐FS‐A vs. GBLUP‐A)

Very few studies have reported ${\hat{h}}^{2}$ values for WRC. For HT growth, Cherry (1995) reported ${\hat{h}}^{2}$ ranging from 0.12 to 0.38 for a small provenance‐progeny test (20 provenances and five families per provenance) for 1‐ to 3‐year‐old seedlings. Given that 73% of our PX females (19 out of 26) were selected for growth, and that low ${\hat{h}}^{2}$ is generally observed for this trait in conifers (Cornelius, 1994), our observations (${\hat{h}}^{2}$ of 0.13 and 0.09 from GBLUP‐A for HT and DBH, respectively) were expected. Other forest tree GBLUP studies reported HT and DBH ${\hat{h}}^{2}$ ranging from zero to 0.25 (Beaulieu et al., 2020; Calleja‐Rodriguez et al., 2020; Gamal El‐Dien et al., 2016, 2018; Lenz, Nadeau, Azaiez et al., 2020; Lenz, Nadeau, Mottet et al., 2020).

For wood extractives, Russell and Daniels (2010) reported ${\hat{h}}^{2}$ estimates ranging from 0.25 to 0.58 for samples collected from a 20‐year‐old clonal trial. They also reported that plicatic acid had the highest coefficient of variation (CV%) of all wood traits (189%). We too found that W.TL, which is the sum of plicatic acid and its lactone, gave the highest CV (115%), when compared to other wood extractives (13%–22%). Russell and Daniels (2010) also found that at a young age (10 years) there were no detectable levels of plicatic acid. They reported genetic correlation of 0.48 between W.AT and plicatic acid at age 20 years, which agrees with our estimated genetic correlation between W.AT and W.TL (0.47, Table 4). In general, conifer terpenoids are associated with resistance to pest, disease, animals, and could be considered as genetic markers. Their inheritance varies from single genes with major effect, to complex quantitative polygenic traits (Hanover, 1992).

Several GBLUP studies for different conifer species, trials and traits (Beaulieu et al., 2020; Calleja‐Rodriguez et al., 2020; Gamal El‐Dien et al., 2016, 2018; Lenz, Nadeau, Azaiez et al., 2020; Lenz, Nadeau, Mottet et al., 2020) have shown that ${\hat{h}}^{2}$ is overestimated by ABLUP when compared to GBLUP, which is consistent with our results for F.AT, F.TM and W.TL. Getting a higher ${\hat{h}}^{2}$ (GBLUP‐A vs. ABLUP‐FS‐A) for W.AT, W.TT, and W.TE, could be due to the fact that the ***G*** matrix might be successful in capturing linkage disequilibrium (LD) between the SNP marker and some QTLs related to these wood chemical traits. Habier et al. (2013) reported that GBLUP not only captured additive relationship, but also LD of SNP with QTL and the co‐segregation to capture relationships at QTL. Another explanation is that captured MST and historical relatedness, given WRC evolutionary history (O'Connell et al., 2008), could be very significant for these traits.

Absence of negative genetic correlations between all traits indicates that multi‐trait genetic gains should be possible with minimal trade‐offs. The high genetic and phenotypic correlation between F.TM and F.AT (0.97) could be explained by the fact that F.AT is the major monoterpene and represents ~58% of the F.TM, as has been observed in other studies (Foster et al., 2016; Kimball et al., 2005; Vourc'h, Russell, & Martin, 2002). W.TT showed a perfect correlation (1.00) with W.TE, as thujaplicins represent around 91% of the W.TE. The strong genetic correlation between thujaplicins and lignans (0.59) is promising for the use of thujaplicins for the indirect selection of the later expressed lignans. The moderate correlation between W.TL and HT and DBH (0.47 and 0.43, respectively), could indicate that faster growing, larger trees might begin producing lignans at earlier ages. However, selection for HT and DBH reduced the expected genetic gain for W.TL from 55% to 26% and 9%, respectively, based on genetic correlations being less than one.

#### 4.2.2 Genotype × environment interaction

All traits (except W.TL) had significant G × E terms with GBLUP‐A, as opposed to ABLUP‐PX. Considering that lignans are expressed at later ages in WRC wood than thujaplicins (Russell & Daniels, 2010), this could explain why we did not see G × E effects. Further studies on older trees are needed to obtain conclusive results on G × E in lignans. As expected, growth traits showed the most G × E. This result could be explained in part by moderate infection by CLB at Jordan River and Port McNeill, as CLB reduces growth rates (Russell & Yanchuk, 2012), and could bias G × E estimates when comparing with the uninfected Powell River site. Similarly in spruce, growth traits have smaller ${\hat{r}}_{B}$ compared to wood quality traits (Lenz, Nadeau, Azaiez et al., 2020; Lenz, Nadeau, Mottet et al., 2020). Given the balanced representation of families across the three sites (on average 20 trees/PX family/site), and that we are using GBLUP, we believe that our estimates are an accurate representation of G × E in WRC.

#### 4.2.3 Dominance genetic effect

We identified significant dominance effects for HT, DBH, and W.TL using the GBLUP‐AD model. Given the small FS family sizes (i.e., 1–15 OTs/FS family, mean = 3.3), further studies in larger FS families are necessary to verify these effects. Despite doubling of heritabilities using the GBLUP‐AD (${\hat{H}}^{2}$) model compared to GBLUP‐A (${\hat{h}}^{2}$), there is a no or a very small decrease in the additive variance estimates (Table 1). This could explain, the perfect correlation between BVs from the two models. Our result suggests that the dominance effect is mainly confounded with the residual variance and not the additive variance. This observed increased heritability could improve genetic gain through clonal deployment. Our result is in agreement with other studies identifying significant dominance effects using GBLUP (Beaulieu et al., 2020; de Almeida Filho et al., 2016; Gamal El‐Dien et al., 2018; Muñoz et al., 2014).

### 4.3 GBLUP advantages for forward and backward selection

#### 4.3.1 ABLUP‐FS‐A and GBLUP‐A increase selection intensity and accuracy for backward selection

The resulting smaller BV $\hat{r}$ for FPTs and MPTs in GBLUP compared to pedigree‐based models was also observed in a similar study using PX in white spruce (Lenz, Nadeau, Azaiez et al., 2020). This may be related to ASReml BV's SE formula, which depends on the number of related individuals. For ABLUP, this number of relationships is calculated from individuals having a constant average value of relationship within family, whereas in GBLUP, relationship values are distributed around this average such that related individuals should have exact relationship coefficient values, resulting in the identification of a smaller number of related individuals. This leads to smaller SE in ABLUP, due to the larger number of identified related individuals, resulting in higher BV theoretical accuracy, which may not be an accurate reflection of true BV accuracy. The parental BV $\hat{r}$ should be more affected than OTs, due to the absence of phenotypes and the smaller number of related individuals (e.g., each parent has on average 58 (FPT) or 68 (MPT) OTs, while each OT has on average 126 HS and 3.3 FS). This could explain why parental BV SE is larger in GBLUP‐A compared to pedigree models, while having the same BV magnitude, which results in lower theoretical accuracy.

Pedigree reconstruction (ABLUP‐FS‐A) resulted in the same average FPTs BV $\hat{r}$ as ABLUP‐PX or a small increase (0.01) for five traits but with more variation reflecting higher variability in the corrected maternal family size (12–68) relative to the original size (38–61). This outcome agrees with results reported in others studies (Doerksen & Herbinger, 2010; Lenz, Nadeau, Azaiez et al., 2020; Vidal et al., 2015, 2017) (Table 3 and Table S4). Given the pedigree correction on the maternal side, ABLUP‐FS‐A and GBLUP‐A resulted in more accurate FPT BVs estimates. ABLUP‐FS‐A and GBLUP‐A analyses gave the opportunity to estimate male BVs with similar $\hat{r}$ (or smaller by 0.01–0.02) to female BVs, resulting in increased selection intensity for backward selection. However, these estimates showed more variation, due to paternal family size variability (7–187) compared to the corrected maternal family size (12–68). This is consistent with a maritime pine PX study (Vidal et al., 2015) which showed substantial variation in paternal BV $\hat{r}$ depending on the number of OTs/MPT.

#### 4.3.2 GBLUP increases BV accuracy and expected genetic gain for forward selection

The feasibility of pedigree reconstruction or GBLUP in PX mating design resulted in increased OT BV accuracy as well as application of more informed forward selection (Bouffier et al., 2019; Doerksen & Herbinger, 2010; Lenz, Nadeau, Azaiez et al., 2020; Vidal et al., 2015, 2017). When comparing GBLUP‐A to ABLUP‐PX, we found that GBLUP‐A increased OT BV accuracy by 17% (for F.AT and W.AT) to 22% (DBH). This improvement was expected as now each OT receives additional information from the paternal side. It is worth mentioning that the largest improvement in BV accuracy is for the least heritable trait, DBH (${\hat{h}}^{2}$ = 0.09 from GBLUP‐A). A similar increase in GBLUP OT BV accuracy of 14% (wood density traits with larger ${\hat{h}}^{2}$) to 25% (growth traits with smaller ${\hat{h}}^{2}$) relative to ABLUP‐PX was reported in white spruce (Lenz, Nadeau, Azaiez et al., 2020). These two studies supported the idea that traits with low ${\hat{h}}^{2}$ will benefit the most from pedigree reconstruction and molecular breeding. In the same study (Lenz, Nadeau, Azaiez et al., 2020), an increase of 4%–7% in OT BV accuracy was observed in the GBLUP‐A model as compared to the ABLUP‐FS‐A model, while in our study we did not find an increase. This could be explained by the larger numbers of related HS for each individual and the sample size in our study (126 HS on average, *N* = 1506) relative to that of the white spruce study (67.6 HS on average, *N* = 892). This may lead us to conclude that when the family size increases, the results from ABLUP approach those of GBLUP. This is further supported by the high Pearson correlation between the BVs from the ABLUP‐FS‐A and GBLUP‐A models (Table S3).

In our study, we selected the top 5% from the 1506 OTs to estimate the expected genetic gain without putting any restriction on genetic diversity; however, this would have to be factored in with real selections in the breeding program. GBLUP‐A greatly improved the expected genetic gain over the PX model by 12% (F.AT) to 35% (W.AT). It should be noted that traits with low ${\hat{h}}^{2}$ (growth and wood traits) showed more genetic gain improvement (i.e., 21% for W.TE, and ranging from 31% to 35% for the other five traits) compared to high ${\hat{h}}^{2}$ foliar traits (12% and 16% for F.AT and Total Foliar, respectively). This again supports the expectation that traits with low ${\hat{h}}^{2}$ will benefit more from molecular breeding.

Given the outperformance of GBLUP‐A over the pedigree models, and that it gives the highest expected gain for all traits, this model was used for comparison of genetic gains. The very high estimate of expected genetic gain for “W.TL”, in spite of its small ${\hat{h}}^{2}$ (0.14), can be justified as “W.TL” showed the highest amount of phenotypic variation with CV of 117%. However, this extremely high CV can be explained partly by the absence of selection for lignans in the study population and the late expression of lignans in the trees' lifespan (Russell & Daniels, 2010). Therefore, while not all trees are expected to express lignans at 18 years, this may still indicate some potential ‘winners’ in future selections. In a study by Beaulieu et al. (2020) in FS white spruce progeny trials (*N* = 598) which examined the levels of acetophenone aglycones (AAs) in needles associated with spruce budworm resistance, a compound called “pungenol” showed a very similar distribution to “W.TL” with CV of 103%. In their study, pungenol showed the highest expected genetic gain of 45.4% due to its high ${\hat{h}}^{2}$ and CV. As expected for foliar traits in our study, given their high ${\hat{h}}^{2}$ across all traits (0.25–0.28) and that only one female of the 26 was selected for foliar traits, showed the second highest expected genetic gain of 22%–25.5%. The other five traits showed a gain range from 6.01% (HT) to 11% (W.TE). These values are expected given their lower ${\hat{h}}^{2}$ and the presence of some background selection for these traits already in the study population. Because no previous studies have reported expected gains in WRC, here we compare expected gain estimates for HT and DBH to two studies in 38 PX (*N* = 892) and 136 FS (*N* = 1516) families in white spruce that used the same gain calculation method (Beaulieu et al., 2020; Lenz, Nadeau, Azaiez et al., 2020). The expected gain in these two studies ranged from 6.36% to 9.35% (${\hat{h}}_{\mathrm{ind}}^{2}$ ranged from 0.13 to 0.25, *N*s (estimated in the PX study only) ranged from 9.43 to 11.85). Here, the gain ranged from 6.01% to 7.44% (${\hat{h}}_{\mathrm{ind}}^{2}$ ranged from 0.09 to 0.13, *N*s ranged from 7.9 to 10.3), similar to those reported before despite the smaller ${\hat{h}}^{2}$ and number of families. In addition to having a different species, these comparable genetic gain estimates may be also due to our greater selection intensity (*N* = 1520 vs. 892 in PX spruce). Beaulieu et al. (2020) also examined foliar chemical traits (*N* = 598), which they found to have higher ${\hat{h}}^{2}$ and expected genetic gain compared to growth traits, affirming the results of our study.

### 4.4 SNPs type and number performance are trait dependent

For genotype proportion, genic‐coding showed the highest heterozygote proportion (27%) while intergenic showed the lowest (23%). The opposite trend is generally expected given the fact that heterozygosity is expected to be higher in non‐coding regions; however, targeted sampling of probe regions with variation results in non‐random sampling and so these estimates do not represent the true distribution of heterozygosity across the genome. Considering the relationship coefficient, it is hard to say if this observed difference across the four ***G*** matrices is significant or not. Given the reported inbreeding in western redcedar from previous studies (Russell et al., 2003; Russell & Ferguson, 2008; Wang & Russel, 2006), ***G***_gen‐no‐cod_ might be the best ***G*** matrix in recovering relatedness, as it gives higher relatedness and inbreeding coefficients compared to other ***G*** matrices.

A comparison of the four GBLUP‐A models in terms of ${\hat{h}}^{2}$ and AIC leads to the conclusion that there is no common pattern across the four ***G*** matrices to identify the best SNP set. This trait‐specific pattern in term of numbers and types of SNPs was observed by Tan et al. (2017) in two Eucalyptus species for GBLUP prediction accuracy (PA), but they did not compare ${\hat{h}}^{2}$. They reported that intergenic SNPs resulted in a slightly better PA for growth traits, which agrees with our results for HT and DBH in term of the resulted highest ${\hat{h}}^{2}$, while genic‐coding and non‐coding SNPs gave better PA for pulp yield. Several studies reported that GS PA reached a plateau by increasing the number of SNPs, which varied from 500 to 15,000 (Chen et al., 2018; Lenz et al., 2017; Tan et al., 2017; Thistlethwaite et al., 2020). We think that the effect on PA will have a mirror effect on ${\hat{h}}^{2}$, which will expectedly reach the same plateau. de Lima et al. (2019) reported that ${\hat{h}}^{2}$ of several traits in Eucalyptus increased with increasing number of SNPs and stabilized when more than 10,000 randomly selected SNPs were used. Given that all differences are very small and the absence of significance testing among our four comparisons, we believe that using all available SNPs is currently the best approach for reducing the sampling variance associated with allele frequency estimates used to calculate the relationship coefficients (Isik et al., 2017).

## 5 CONCLUSIONS

- Pedigree reconstruction confirmed the expected unequal male contribution in the PX mating design and revealed unexpected maternal errors, improving the pedigree files in the analysis. Accounting for the missing paternal information has small or no effect on the FPTs' BV accuracy but led to significant improvement in OT's BV accuracy. This effect was stronger in low heritability traits. In general, PX mating design will benefit the most from pedigree reconstruction or genomic analysis compared to other designs.
- Genomic analysis (GBLUP‐A) overcame the limitation of PX mating design for forward selection, increased selection intensity and accuracy for backward selection, and increased the expected genetic gain. Moreover, it eliminates the need of pedigree reconstruction, all of which increases breeding efficiency.
- Considering that our study population contains a degree of PX family selection for growth and wood traits, we can conclude that a selected advanced breeding population, accompanied with reduced genetic diversity, and low heritability traits in general could benefit more from molecular breeding in future generations.
- In summary, all traits show low to medium genetic control, acceptable levels of G × E, and no evidence of trade‐off in the expected genetic gain from the three traits (growth, foliar and heartwood) categories. This calls for more studies to investigate the possibility of using growth and total thujaplicins for the indirect selection of the later expressed lignans. As indicated earlier, GS techniques for predicting genetic gains in later‐expressed wood quality traits, such as that of thujaplicins and lignans, would be a cost‐effective approach for improving such traits, because they are difficult to be selected for in the normal time frame of tree breeding operations. Operationally, the next step would be to perform different multi‐trait selection scenarios using the selection index, or independent culling scenarios and look for trees with potential positive gains in all traits. This will result in the greater yield of these secondary metabolites responsible for deer browsing and heartwood rot resistance, as well as faster growing trees. It is worth mentioning that applying GS for multi‐trait early selection operationally in the WRC breeding program, at the seedling phase, is currently in progress.

## CONFLICT OF INTEREST

The authors declare that they have no conflict of interest

## Supporting information


Appendix S1

Table S1

Figure S1

## Data Availability

SNP genotyping data are available on Zenodo, and can be assessed at https://zenodo.org/record/6562381#.YqOw9nbMKUl. The phenotypic data are stored in the institutional database of BC WRC breeding program and will be shared upon request to the corresponding author or the project leaders (http://futurecedarforests.ca/), in compliance with the BC Government Intellectual Property Policies.
