# Supplementary material for: Genomic selection reveals hidden relatedness and increased breeding efficiency in western redcedar polycross breeding
Source: Evol Appl. 2022 Aug 23;15(8):1291–312. doi: 10.1111/eva.13463 (PMC9423091; doi:10.1111/eva.13463)
Supplement: Supplementary file 1 — Appendix S1 [file EVA-15-1291-s003.docx]

**Appendix 1:**

1. **Abbreviations**

| **Abbreviations** | |
| --- | --- |
| **Breeding** | |
| BC | British Columbia |
| WRC | western redcedar |
| FPT | female parent tree |
| MPT | male parent tree |
| OT | offspring tree |
| FS | full-sibs |
| HS | half-sibs |
| OP | open pollinated |
| PX | polycross |
| **Genotyping** | |
| AB | allele balance |
| EM | Expectation-maximization |
| LD | linkage disequilibrium |
| MAF | minor allele frequency |
| SNP | Single Nucleotide Polymorphic |
| **Phenotyping** | |
| HT | height |
| DBH | diameter at breast heigh |
| F.TM | foliar total monoterpenes |
| F.AT | foliar α-thujone |
| W.TT | wood total thujaplicins |
| W.AT | wood α-thujaplicin |
| W.TL | wood total lignans |
| W.TE | wood total extractives |
| CLB | cedar leaf blight |
| CWW | conditioned wood weight |
| DFW | dry foliar weight |
| GC | gas chromatography |
| FID | flame ionization detector |
| MS | mass spectrometry |
| **Models' estimates** | |
| ABLUP | traditional pedigree-based Best Linear Unbiased Prediction analysis |
| GBLUP | Genomic-based Best Linear Unbiased Prediction analysis |
| GS | Genomic Selection |
| AIC | Akaike Information Criterion |
| BV | breeding value |
| CV | coefficient of variation |
| G × E | genotype × environment |
| *h^^2^* | narrow-sense heritability |
| *H^^2^* | broad-sense heritability |
| *Ns* | status number |
| *r ̂* | theoretical accuracy |
| *r^^^_B_* | type-B additive genetic correlation |
| LRT | likelihood ratio test |
| SD | standard deviation |

1. **Additive effect model:**

$\boldsymbol{y}=\boldsymbol{X\beta}+\boldsymbol{Z}_{\mathbf{1}}\boldsymbol{r}\left( \boldsymbol{s} \right)+\boldsymbol{Z}_{\mathbf{2}}\boldsymbol{ib}\left( \boldsymbol{r} \right)+ \boldsymbol{Z}_{\mathbf{3}}\boldsymbol{a}+\boldsymbol{Z}_{\mathbf{4}}\boldsymbol{sa}+\boldsymbol{e}$ **(1)**

where $\boldsymbol{y}$ is the vector of measured phenotypes; $\boldsymbol{\beta}$ are the vectors of the fixed effects including site means, and heartwood width (mm) and total number of rings (the sum of measured and estimated number of missed rings) used as covariates for wood traits only; $\boldsymbol{r}\left( \boldsymbol{s} \right)$ is the vector of random replicate (block) nested within site effect, following $var \left( \boldsymbol{r}\left( \boldsymbol{s} \right) \right)\sim N\left( 0,{\boldsymbol{I}\sigma}_{r\left( s \right)}^{2} \right)$; $\boldsymbol{ib}\left( \boldsymbol{r} \right)$ is the vector of random incomplete block (set) nested within replicate (block) effect, following $var \left( \boldsymbol{ib}\left( \boldsymbol{r} \right) \right)\sim N\left( 0, {\boldsymbol{I}\sigma}_{ib\left( r \right)}^{2} \right)$; $\boldsymbol{a}$ is the vector of random additive genetic effect (breeding values), following $var \left( \boldsymbol{a} \right)\sim N\left( 0,{\boldsymbol{A}_{\boldsymbol{PX}}\sigma}_{a}^{2} \right)$ for the ABLUP-PX model, $var \left( \boldsymbol{a} \right)\sim N\left( 0,{\boldsymbol{A}_{\boldsymbol{FS}}\sigma}_{a}^{2} \right)$ for the ABLUP-FS-A model, and $var \left( \boldsymbol{a} \right)\sim N\left( 0,{\boldsymbol{G}_{\boldsymbol{all}} \sigma}_{a}^{2} \right)$ for the GBLUP-A model; $\boldsymbol{sa}$ is the vector of random interaction of site with additive genetic effects, following $var\left( \boldsymbol{sa} \right)\sim N\left( 0,\boldsymbol{A}_{\boldsymbol{PX}}\otimes\boldsymbol{I}\sigma_{sa}^{2} \right)$ for the ABLUP-PX model, $var\left( \boldsymbol{sa} \right)\sim N\left( 0,\boldsymbol{A}_{\boldsymbol{FS}}\otimes\boldsymbol{I}\sigma_{sa}^{2} \right)$ for the ABLUP-FS-A model, $var\left( \boldsymbol{sa} \right)\sim N\left( 0,\boldsymbol{G}_{\boldsymbol{all}}\otimes\boldsymbol{I}\sigma_{sa}^{2} \right)$ for the GBLUP-A model, where ⊗ represents the Kronecker product of matrices; and $\boldsymbol{e}$ is the vector of random heterogenous residuals, following $var \left( \boldsymbol{e} \right)\sim N\left( 0,\boldsymbol{R} \right)$, where ***R*** is a block diagonal matrix $R= \bigoplus I\sigma_{e}^{2}$, $\sigma_{e}^{2}$ is the site-specific residual variance and ⨁ is the “direct sum” of site-specific residual matrices. ***A_PX_***, ***A_FS_*** and ***G_all_*** are additive relationship matrices from PX-pedigree, FS-pedigree and genomic SNP markers, respectively. $\sigma_{ib\left( r \right)}^{2},\sigma_{r\left( s \right)}^{2}, \sigma_{a}^{2}, \sigma_{sa}^{2}$ and $\sigma_{e}^{2}$ are incomplete block (set) nested within replicate, replicate (block) nested within site, additive genetic, interaction of site with additive genetic, and residual variances, respectively. ***X*** and ***Zs*** are incidence matrices relating the phenotypes to the model terms and ***I*** is the identity matrix with the proper dimension.

1. **Additive and dominance effect model:**

$\boldsymbol{y}=\boldsymbol{X\beta}+\boldsymbol{Z}_{\mathbf{1}}\boldsymbol{r}\left( \boldsymbol{s} \right)+\boldsymbol{Z}_{\mathbf{2}}\boldsymbol{ib}\left( \boldsymbol{r} \right)+ \boldsymbol{Z}_{\mathbf{3}}\boldsymbol{a}+\boldsymbol{Z}_{\mathbf{4}}\boldsymbol{sa}+\boldsymbol{Z}_{\mathbf{5}}\boldsymbol{d}+\boldsymbol{Z}_{\mathbf{6}}\boldsymbol{sd}+\boldsymbol{e}$ **(2)**

where $\boldsymbol{d}$ is the vector of random dominance genetic effects, following $var \left( \boldsymbol{d} \right)\sim N\left( 0,{\boldsymbol{A}_{\boldsymbol{d}}\sigma}_{d}^{2} \right)$ for the ABLUP-FS-AD model and $var \left( \boldsymbol{d} \right)\sim N\left( 0,{\boldsymbol{G}_{\boldsymbol{d}}\sigma}_{d}^{2} \right)$ for the GBLUP-AD model; and $\boldsymbol{sd}$ is the vector of random interaction of site with dominance genetic effects, following $var\left( \boldsymbol{sd} \right)\sim N\left( 0,\boldsymbol{A}_{\boldsymbol{FS}}\otimes\boldsymbol{I}\sigma_{sd}^{2} \right)$ for the ABLUP-FS-AD model, $var\left( \boldsymbol{sd} \right)\sim N\left( 0,\boldsymbol{G}_{\boldsymbol{d}}\otimes\boldsymbol{I}\sigma_{sd}^{2} \right)$ for the GBLUP-AD model. ***A_d_*** and ***G_d_*** are dominance relationship matrices from FS-pedigree and genomic SNP markers, respectively. $\sigma_{d}^{2}$ and $\sigma_{sd}^{2}$ are dominance genetic and interaction of site with dominance genetic variances, respectively. The others terms are as defined in Equation 1.

1. **Heritability and type-B genetic correlation**

The narrow-sense heritability ($\hat{h}^{2}$), broad-sense heritability ($\hat{H}^{2}$), and type-B additive genetic correlation ($\hat{r}_{B})$ for additive genetic effect, which measures the magnitude of genotype × environment (G × E) interaction, were estimated as follow:

$\hat{h}^{2}=\frac{\hat{\sigma}_{a}^{2}}{\hat{\sigma}_{a}^{2}+\hat{\sigma}_{sa}^{2}+\hat{\sigma}_{e}^{2}}$ for additive models from Equation 1

$\hat{h}^{2}=\frac{\hat{\sigma}_{a}^{2}}{\hat{\sigma}_{a}^{2}+\hat{\sigma}_{sa}^{2}+\hat{\sigma}_{d}^{2}+\hat{\sigma}_{sd}^{2}+\hat{\sigma}_{e}^{2}}$ for additive-dominance models from Equation 2

$\hat{H}^{2}=\frac{\hat{\sigma}_{a}^{2}+\hat{\sigma}_{d}^{2}}{\hat{\sigma}_{a}^{2}+\hat{\sigma}_{sa}^{2}+\hat{\sigma}_{d}^{2}+\hat{\sigma}_{sd}^{2}+\hat{\sigma}_{e}^{2}}$ for additive-dominance models from Equation 2

$\hat{r}_{B}=\frac{\hat{\sigma}_{a}^{2}}{\hat{\sigma}_{a}^{2}+\hat{\sigma}_{sa}^{2}}$ for models from Equations 1 and 2

where $\hat{\sigma}_{e}^{2}$ is the average of the three sites’ individual $\hat{\sigma}_{e}^{2}.$Standard errors (SE) of these parameters were estimated using the delta method of Venables and Ripley (2000), as implemented in the “vpredict” function from the R package asreml.

1. **The multivariate model**

The following multi-variate model was fitted as follows:

$$\left[ \begin{aligned} \begin{aligned} \begin{matrix} \boldsymbol{y}_{\mathbf{1}} \\ . \end{matrix} \\ . \end{aligned} \\ \boldsymbol{y}_{\boldsymbol{n}} \end{aligned} \right]=\boldsymbol{X\beta}\left( \boldsymbol{t} \right) +\boldsymbol{Z}_{\mathbf{1}}\boldsymbol{r}_{(\boldsymbol{s})}\left( \boldsymbol{t} \right)+\boldsymbol{Z}_{\mathbf{2}}\boldsymbol{ib}_{(\boldsymbol{r})}\left( \boldsymbol{t} \right)+ \boldsymbol{Z}_{\mathbf{3}}\boldsymbol{a}\left( \boldsymbol{t} \right)+\boldsymbol{Z}_{\mathbf{4}}\boldsymbol{sa}\left( \boldsymbol{t} \right)+\boldsymbol{e}(\boldsymbol{t})$$

where ***n*** is the number of traits (2, 3 or 5), ***y_s_*** are the stacked vectors of the n traits’ measured phenotypes, and the model terms are as explained in Equation 1 but nested within trait $\left( \boldsymbol{t} \right)$. $\boldsymbol{\beta}$ are the vector of the fixed effects including each trait mean, site mean within trait, and heartwood width (mm) and total number of rings (the sum of measured and estimated number of missed rings) are used as covariates for wood traits only; $\boldsymbol{ib}_{\boldsymbol{(r)}}\left( \boldsymbol{t} \right)$ is the vector of random incomplete block (set) nested within replicate effect within trait, following $var \left( \boldsymbol{ib}_{\boldsymbol{(r)}}\left( \boldsymbol{t} \right) \right)\sim N\left( 0, {\boldsymbol{I}\otimes V}_{ib\left( r \right)} \right)$; $\boldsymbol{r}_{\boldsymbol{(s)}}\left( \boldsymbol{t} \right)$ is the vector of random replicate nested within site effect within trait, following $var \left( \boldsymbol{r}_{\boldsymbol{(s)}}\left( \boldsymbol{t} \right) \right) \sim N\left( 0,{\boldsymbol{I}\otimes V}_{r\left( s \right)} \right)$; $\boldsymbol{a}\left( \boldsymbol{t} \right)$ is the vector of random additive genetic effect (breeding values) nested within trait, following $var \left( \boldsymbol{a}\left( \boldsymbol{t} \right) \right)\sim N\left( 0,{\boldsymbol{G}\otimes V}_{a} \right)$; $\boldsymbol{sa}\left( \boldsymbol{t} \right)$ is the vector of random interaction of site with additive effects nested within trait, following $var\left( \boldsymbol{sa (t)} \right)\sim N\left( 0,\boldsymbol{I}{\otimes V}_{sa} \right)$; and $\boldsymbol{e}\left( \boldsymbol{t} \right)$ is the vector of random homogeneous (across sites) residual nested within trait, following $var \left( \boldsymbol{e}\left( \boldsymbol{t} \right) \right)\sim N\left( 0,{\boldsymbol{I}\otimes V}_{e} \right)$. The matrices $\boldsymbol{V}_{\boldsymbol{a}}$ and $\boldsymbol{V}_{\boldsymbol{e}}$ are n × n variance-covariance matrices using the CORGH variance structure in ASReml (heterogenous variance and correlation between traits). The matrices ${\boldsymbol{V}_{\boldsymbol{r}\left( \boldsymbol{s} \right)}\boldsymbol{, V}}_{\boldsymbol{ib}\left( \boldsymbol{r} \right)}$**,** and $\boldsymbol{V}_{\boldsymbol{sa}}$ are n × n variance-covariance matrices using the IDH variance structure in ASReml (heterogenous variance and no correlation between traits). The significance of the genetic correlation was estimated by looking into their SE and considering the correlation significant when the ratio of correlation/SE ≥ 2.
