# Supplementary material for: Genomic selection reveals hidden relatedness and increased breeding efficiency in western redcedar polycross breeding
Source: Evol Appl. 2022 Aug 23;15(8):1291–312. doi: 10.1111/eva.13463 (PMC9423091; doi:10.1111/eva.13463)
Supplement: Supplementary file 2 — Table S1 [file EVA-15-1291-s001.docx]

**Table S1** Phenotypic means, standard deviations (SD), and coefficients of variation (CV) across the three sites for tested traits.

| **Trait^a^** | **Mean** | **SD** | **CV** |
| --- | --- | --- | --- |
| **HT** (cm) | 848 | 216 | 25% |
| **DBH** (mm) | 118 | 34 | 29% |
| **F.AT** (µg/g DFW) | 23237 | 7557 | 33% |
| **F.TM** (µg/g DFW) | 39955 | 12906 | 32% |
| **W.AT (**µg/g CWW)**^b^** | 5.7 | 0.94 | 17% |
| **W.TT (**µg/g CWW)**^b^** | 6.7 | 0.88 | 13% |
| **W.TL** (PAR/g CWW)**^b^** | 0.81 | 0.94 | 117% |
| **W.TE** (PAR/g CWW)**^b^** | 3.5 | 0.76 | 22% |

Abbreviations: HT, height; DBH, diameter at breast height; F.AT, foliar α-thujone; F.TM, foliar total monoterpenes; W.AT, wood α-thujaplicin; W.TT, wood total thujaplicins; W.TL, wood total lignans; W.TE, wood total extractives; DFW, dry foliar weight (dried at 70°C); PAR, peak area ratio; CWW, conditioned wood weight (dried at 40°C).

**^a^**1520 trees were phenotyped for growth and foliar chemistry at age 15 years, while only 1510 were phenotyped for wood traits at age 18 years (ten dead or decayed trees in the trial were not sampled).

**^b^**This summary is for the log transformation; we transformed the wood data to meet the normality assumption.**Table S2.** Summary statistics for genomic relationships (***G _all_***) across different relationship classes.

| **Genomic Relationship** | | **Min** | **Max** | **Mean** |
| --- | --- | --- | --- | --- |
| Parent-Offspring | Female-Not selfed offspring | 0.17 | 0.51 | 0.39 |
|  | Female-Selefed offspring | 0.67 | 0.89 |  |
|  | Male | 0.18 | 0.59 | 0.40 |
| Offspring-Offspring (HS) | Maternal HS family | 0.09 | 0.30 | 0.20 |
|  | Paternal HS family | 0.08 | 0.43 | 0.20 |
| Diagonal | Not selfed offspring | 0.72 | 1.54 | 1.11 |
|  | Selefed offspring | 1.48 | 1.57 |  |

**Table S3** Correlation between offspring breeding values from selected models for all tested traits.

| **Models** | **ABLUP-PX**  **ABLUP-FS-A** | **ABLUP-PX**  **GBLUP-A** | **ABLUP-FS-A**  **GBLUP-A** | **ABLUP-FS-A**  **ABLUP-FS-AD** | **GBLUP-A**  **GBLUP-AD** |
| --- | --- | --- | --- | --- | --- |
| **Trait^a^** |  |  |  |  |  |
| **HT** | 0.76 | 0.76 | 0.94 | 1.00 | 1.00 |
| **DBH** | 0.74 | 0.74 | 0.93 | 1.00 | 1.00 |
| **F.AT** | 0.84 | 0.82 | 0.96 | 1.00 | 1.00 |
| **F.TM** | 0.81 | 0.79 | 0.96 | 1.00 | 1.00 |
| **W.AT** | 0.79 | 0.82 | 0.92 | 1.00 | 1.00 |
| **W.TT** | 0.76 | 0.79 | 0.91 | 1.00 | 1.00 |
| **W.TL** | 0.72 | 0.71 | 0.94 | 1.00 | 1.00 |
| **W.TE** | 0.78 | 0.80 | 0.93 | 1.00 | 1.00 |

**^a^**See Table 1 for traits description.

**Table S4.** Summary statistics for BV theoretical accuracy ($\hat{r})$ of female and male parents from selected models for all tested traits.

| **Traits** | **Model** | **Parents** | **Min.** | **Mean** | **Max.** |
| --- | --- | --- | --- | --- | --- |
| **HT** | ABLUP-PX | females | 0.67 | 0.74 | 0.75 |
|  | ABLUP-FS-A | females | 0.59 | 0.74 | 0.84 |
|  |  | males | 0.43 | 0.73 | 0.84 |
|  | GBLUP-A | females | 0.51 | 0.63 | 0.74 |
|  |  | males | 0.41 | 0.63 | 0.74 |
| **DBH** | ABLUP-PX | females | 0.59 | 0.67 | 0.68 |
|  | ABLUP-FS-A | females | 0.51 | 0.67 | 0.78 |
|  |  | males | 0.36 | 0.66 | 0.78 |
|  | GBLUP-A | females | 0.45 | 0.56 | 0.68 |
|  |  | males | 0.34 | 0.56 | 0.68 |
| **F.AT** | ABLUP-PX | females | 0.79 | 0.84 | 0.85 |
|  | ABLUP-FS-A | females | 0.73 | 0.85 | 0.91 |
|  |  | males | 0.55 | 0.83 | 0.91 |
|  | GBLUP-A | females | 0.62 | 0.72 | 0.80 |
|  |  | males | 0.51 | 0.71 | 0.80 |
| **F.TM** | ABLUP-PX | females | 0.77 | 0.82 | 0.83 |
|  | ABLUP-FS-A | females | 0.70 | 0.83 | 0.90 |
|  |  | males | 0.52 | 0.81 | 0.90 |
|  | GBLUP-A | females | 0.60 | 0.70 | 0.79 |
|  |  | males | 0.48 | 0.70 | 0.79 |
| **W.AT** | ABLUP-PX | females | 0.74 | 0.80 | 0.80 |
|  | ABLUP-FS-A | females | 0.67 | 0.80 | 0.88 |
|  |  | males | 0.47 | 0.78 | 0.88 |
|  | GBLUP-A | females | 0.58 | 0.68 | 0.77 |
|  |  | males | 0.45 | 0.67 | 0.77 |
| **W.TT** | ABLUP-PX | females | 0.73 | 0.78 | 0.79 |
|  | ABLUP-FS-A | females | 0.66 | 0.79 | 0.87 |
|  |  | males | 0.46 | 0.77 | 0.87 |
|  | GBLUP-A | females | 0.57 | 0.67 | 0.76 |
|  |  | males | 0.44 | 0.66 | 0.76 |
| **W.TL** | ABLUP-PX | females | 0.73 | 0.80 | 0.81 |
|  | ABLUP-FS-A | females | 0.63 | 0.80 | 0.91 |
|  |  | males | 0.45 | 0.78 | 0.91 |
|  | GBLUP-A | females | 0.55 | 0.68 | 0.80 |
|  |  | males | 0.43 | 0.67 | 0.80 |
| **W.TE** | ABLUP-PX | females | 0.74 | 0.80 | 0.80 |
|  | ABLUP-FS-A | females | 0.67 | 0.80 | 0.87 |
|  |  | males | 0.49 | 0.78 | 0.87 |
|  | GBLUP-A | females | 0.58 | 0.68 | 0.77 |
|  |  | males | 0.46 | 0.67 | 0.77 |

**Table S5** Percentage of overestimation of expected genetic gain and *Ns* from the pedigree additive models (ABLUP-PX and ABLUP-FS-A) compared to GBLUP-A, and increase in BV theoretical accuracy and expected genetic gain from GBLUP-A compared to pedigree additive models for all tested traits. This comparison is for the selected top 5% trees (Census Number *=*75) using the genetic gain estimates in Table 4.

| **Trait^a^** | **Model** | **Pedigree additive models** | | | **GBLUP-A** | | |
| --- | --- | --- | --- | --- | --- | --- | --- |
|  |  | **Gain overestimation %^b^** | | **Ns** **overestimation %^c^** | | **Increase in accuracy %^d^** | **Increase in gain %^e^** |
| **HT** | **ABLUP-PX** | 27 | 29 | | 19 | | 34 |
|  | **ABLUP-FS-A** | 13 | --- | | -5 | | 13 |
| **DBH** | **ABLUP-PX** | 14 | 12 | | 22 | | 31 |
|  | **ABLUP-FS-A** | 0 | --- | | -6 | | 9 |
| **F.AT** | **ABLUP-PX** | 2 | 41 | | 17 | | 12 |
|  | **ABLUP-FS-A** | 8 | --- | | 1 | | 5 |
| **F.TM** | **ABLUP-PX** | -5 | 44 | | 18 | | 16 |
|  | **ABLUP-FS-A** | 10 | --- | | 0 | | 5 |
| **W.AT** | **ABLUP-PX** | 51 | 17 | | 17 | | 35 |
|  | **ABLUP-FS-A** | -3 | --- | | -2 | | 12 |
| **W.TT** | **ABLUP-PX** | 24 | 22 | | 19 | | 31 |
|  | **ABLUP-FS-A** | -5 | --- | | -2 | | 9 |
| **W.TL** | **ABLUP-PX** | 20 | 1 | | 20 | | 34 |
|  | **ABLUP-FS-A** | 4 | --- | | -3 | | 6 |
| **W.TE** | **ABLUP-PX** | 32 | 35 | | 19 | | 21 |
|  | **ABLUP-FS-A** | 3 | --- | | -2 | | 3 |

**^a^**See Table 1 for traits description.

**^b^**Gain overestimation % = ([Gain % estimated from ABLUP - Corrected gain % estimated from GBLUP-A]/Corrected gain % estimated from GBLUP-A) *100.

**^c^**Ns overestimation % = ([Ns (from PX pedigree) - Ns (from FS pedigree)]/ Ns (from FS pedigree)) *100. Ns is the status number of the selected top 75 trees and used as a measure for the genetic diversity.

**^d^**Increase in accuracy % = (Theoretical BVs’ accuracy from GBLUP-A - Theoretical BV’s accuracy from ABLUP-PX / Theoretical BV’s accuracy from ABLUP-PX) *100.

**^e^**Increase in gain % = (Gain % from GBLUP-A - Corrected gain % from ABLUP-PX / Corrected gain % from ABLUP-PX) *100.

**Table S6** Comparison between the four genotypes files (all, genic-coding, genic-no-coding and intergenic SNPs) using genotypes proportion for -1,0, and 1.

| **SNPs** | **Number of SNPs** | **Genotype Proportion** | | |
| --- | --- | --- | --- | --- |
|  |  | **-1** | **0** | **1** |
| **All** | 45378 | 62 | 25 | 13 |
| **Gen-cod** | 14767 | 59 | 27 | 14 |
| **Gen-no-cod** | 14406 | 61 | 25 | 14 |
| **Intergen** | 12858 | 66 | 23 | 12 |

Abbreviations: Gen-cod, genic-coding; Gen-no-cod, genic-no-coding; Intergen, intergenic

**Table S7** Comparison between the expected average relationship from FS- and PX- pedigrees, and the average of realized genomic relationship using different ***G*** matrices from the four SNPs files (all, genic-coding, genic-no-coding and intergenic SNPs).

| ***A _FS_*** | ***A _PX_*** | ***G _all_*** | ***G _gen-cod_*** | ***G _gen-no-cod_*** | ***G _intergen_*** |
| --- | --- | --- | --- | --- | --- |
| **0** | 0.00 | -0.02 | -0.02 | -0.02 | -0.02 |
| **0.25** | 0.07 | 0.19 | 0.19 | 0.20 | 0.18 |
| **0.5** | 0.19 | 0.41 | 0.42 | 0.44 | 0.39 |
| **1** | 0.54 | 1.05 | 1.04 | 1.10 | 1.04 |
| **1.5** | 1.00 | 1.55 | 1.53 | 1.61 | 1.54 |

Abbreviations: *A*, pedigree relationship matrix; *G*, Genomic relationship matrix from SNPs; FS, full-sib pedigree; PX, polycross-pedigree; gen-cod, genic-coding; gen-no-cod, genic-no-coding; intergen, intergenic

**Table S8**. Pearson and Spearman correlations between females BVs from ABLUP-PX and ABLUP-FS-A for all tested traits.

| Traits | Pearson | Spearman |
| --- | --- | --- |
| **HT** | 0.87 | 0.84 |
| **DBH** | 0.85 | 0.80 |
| **F.AT** | 0.97 | 0.96 |
| **F.TM** | 0.95 | 0.96 |
| **W.AT** | 0.94 | 0.94 |
| **W.TT** | 0.88 | 0.86 |
| **W.TL** | 0.92 | 0.88 |
| **W.TE** | 0.88 | 0.87 |
