# Supplementary material for: Genomic selection reveals hidden relatedness and increased breeding efficiency in western redcedar polycross breeding
Source: Evol Appl. 2022 Aug 23;15(8):1291–312. doi: 10.1111/eva.13463 (PMC9423091; doi:10.1111/eva.13463)
Supplement: Supplementary file 3 — Figure S1 [file EVA-15-1291-s002.docx]

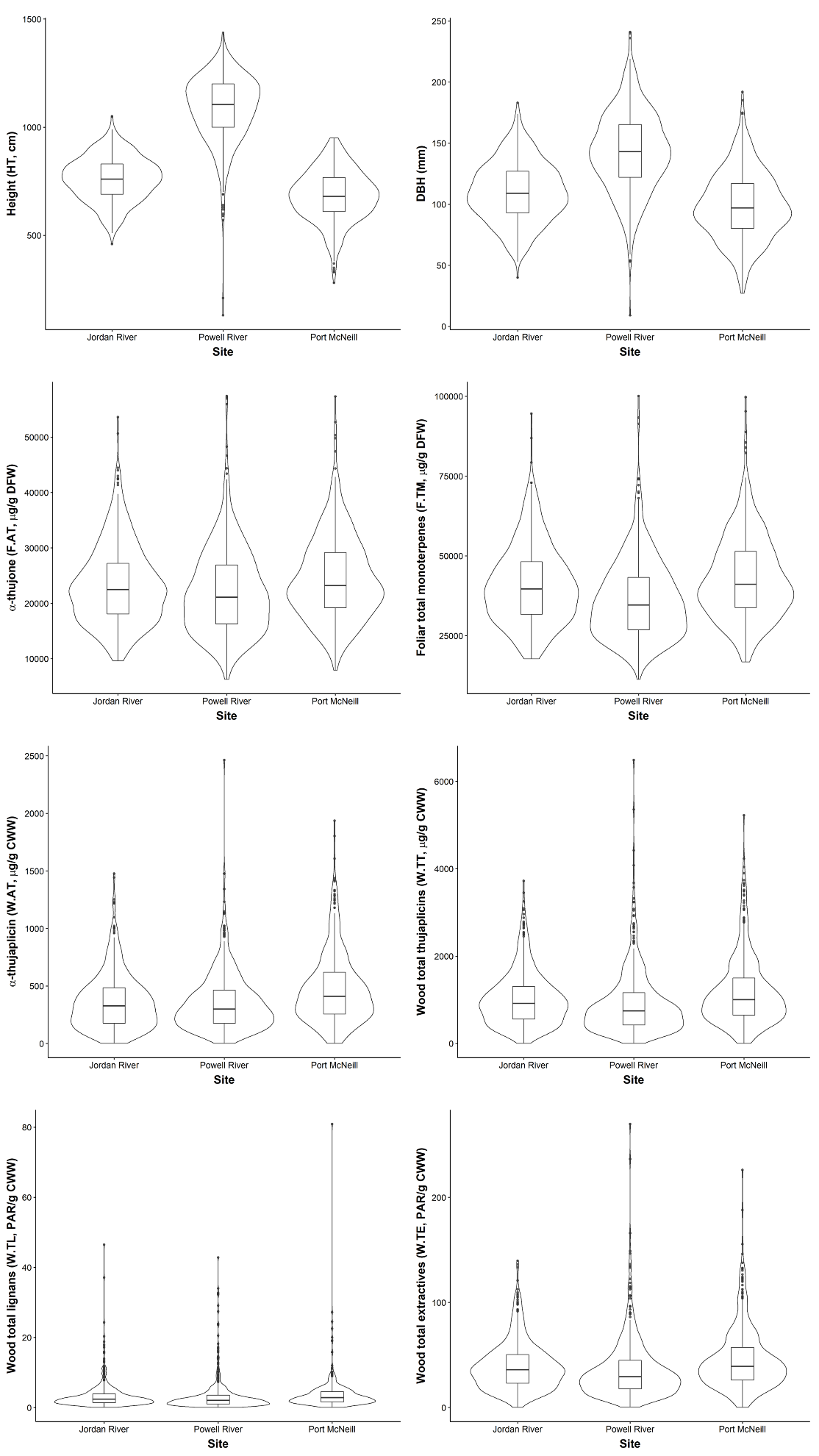


**FIGURE S1** **Box and violin plots grouped by sites for all traits**. Plots show variation within and between sites. Abbreviations: DBH, diameter at breast height; DFW, wet foliar weight; PAR, peak area ratio; CWW, conditioned wood weight (dried at 40°C)


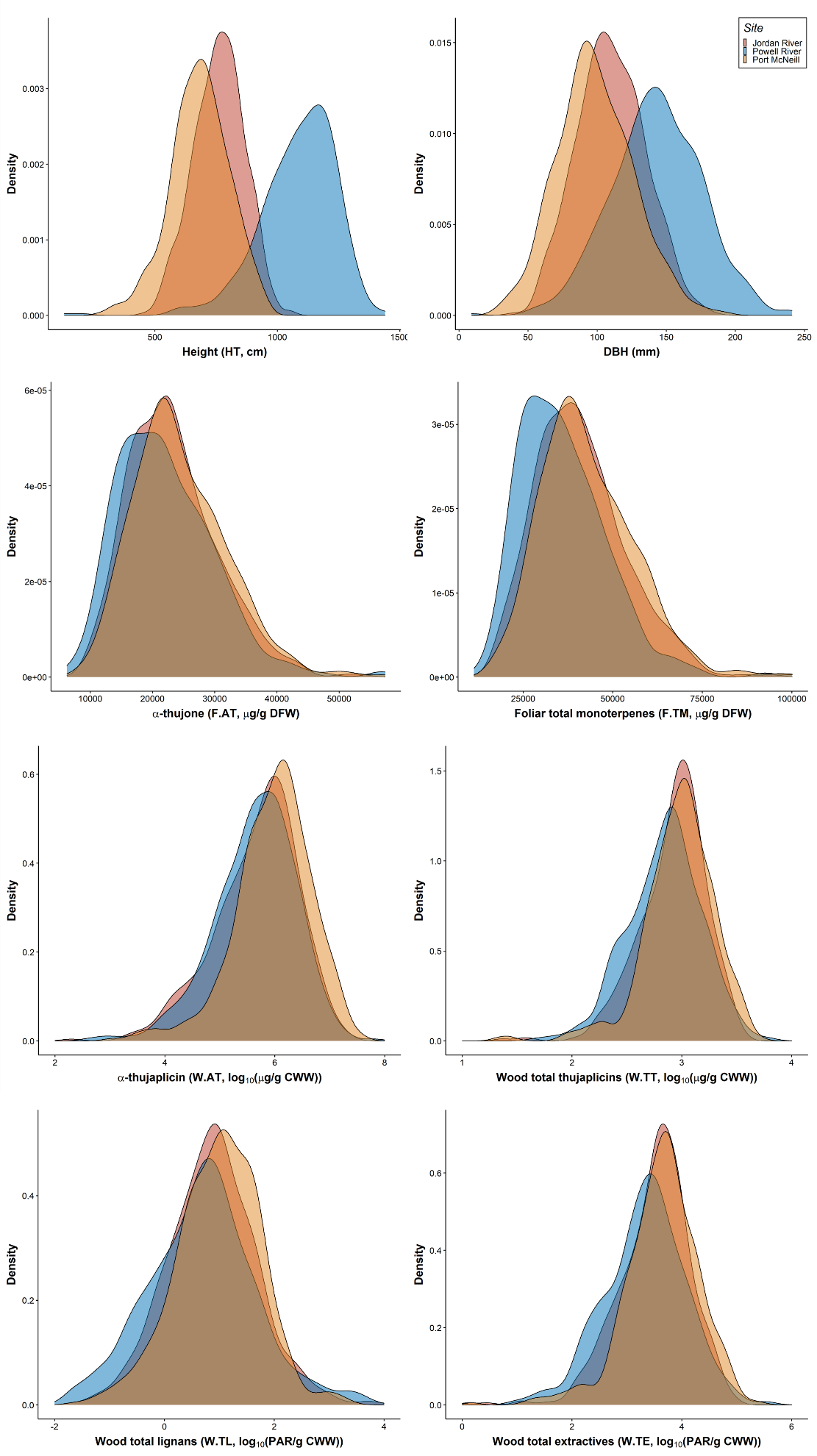


**FIGURE S2** **Density plot of phenotypic distribution for all traits per site**. Wood traits showed a non-normal distribution, so log transformation was used to meet the normality assumption.

Abbreviations: DBH, diameter at breast height; DWF, wet foliar weight; PAR, peak area ratio; CWW, conditioned wood weight (dried at 40°C)


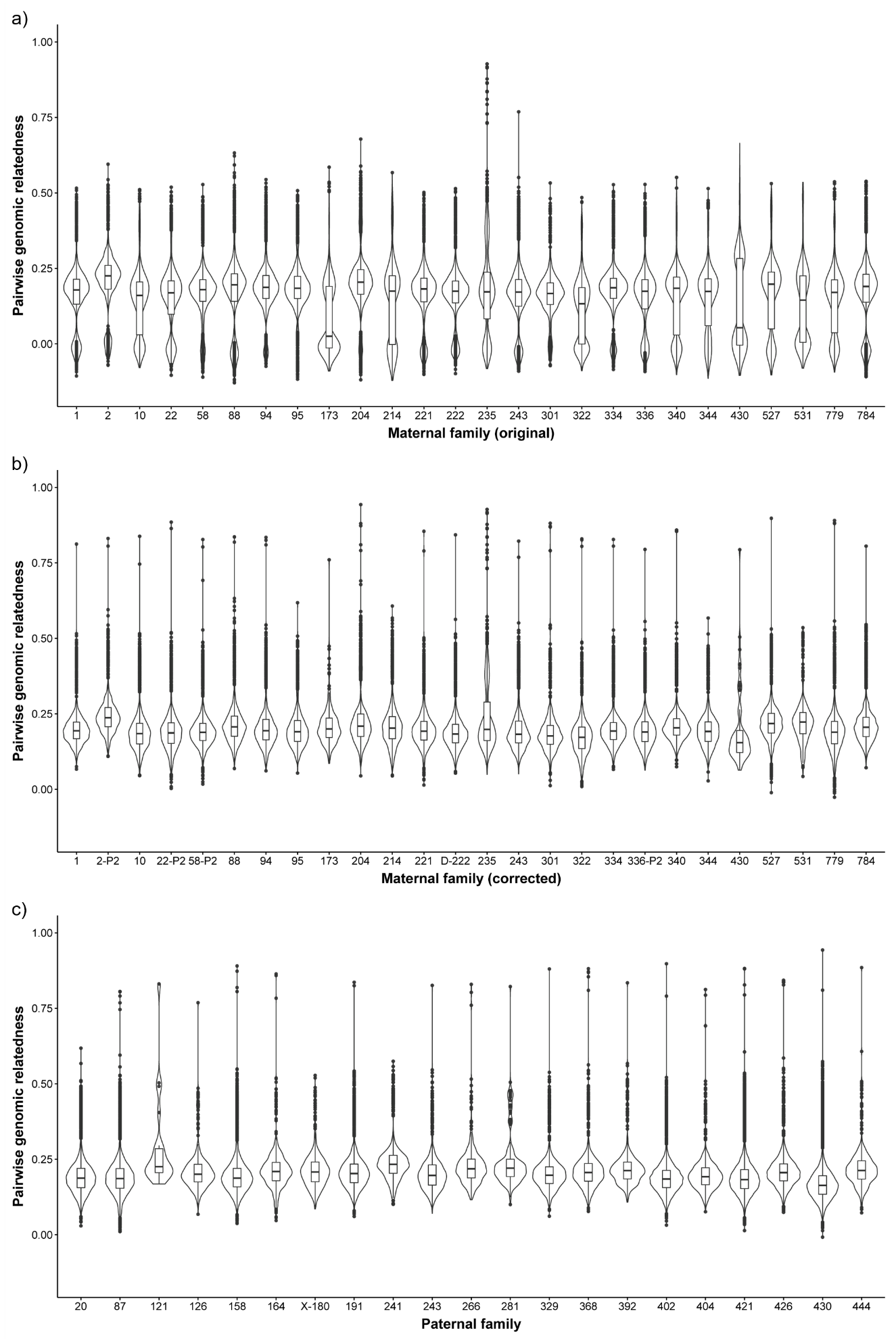


**FIGURE S3** **Box and violin plots of pairwise genomic relationship (*G* matrix) within each family.** (a) Within the original 26 PX maternal families we observe many relationships around 0. (b) Within the corrected 26 PX maternal families we observe no relationship around 0. (c) Within the assigned 21 paternal families we observe no relationship around 0.

*Note*: relationships around 0.5 reflect full-sib and larger values are due to the presence of some self individuals


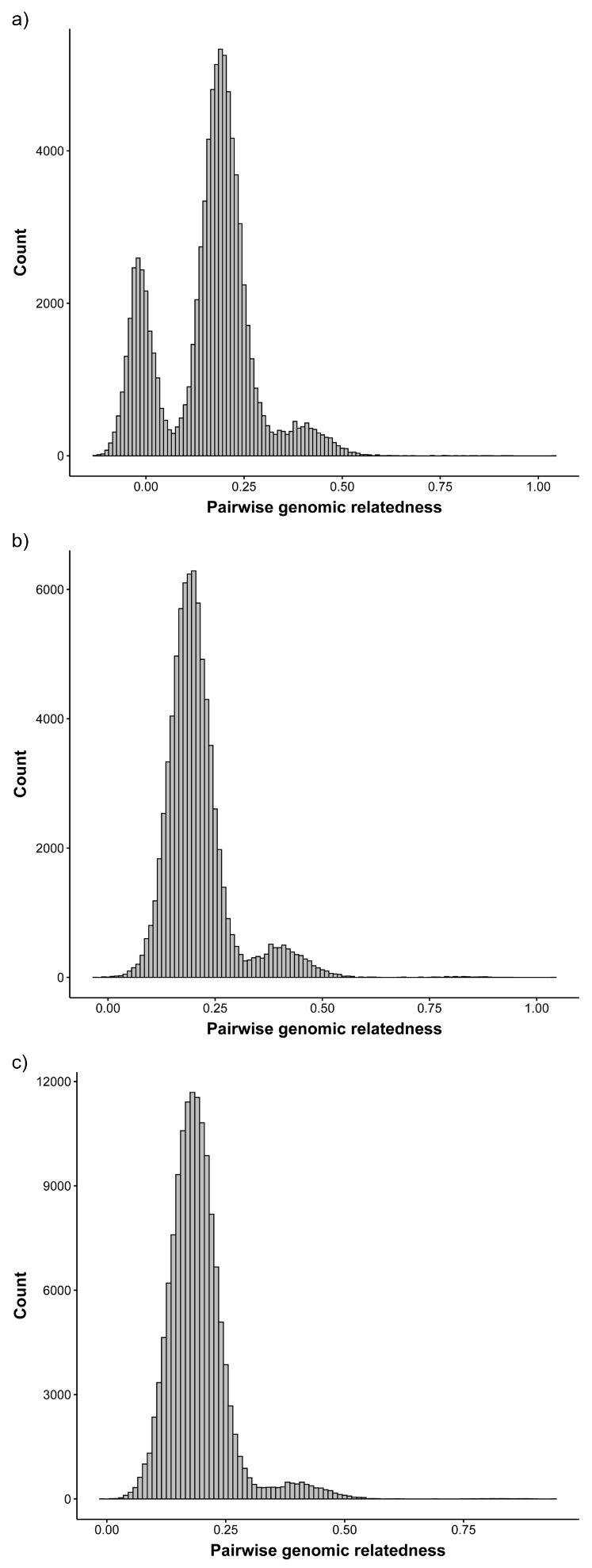


**FIGURE S4** **Histogram of pairwise genomic relationship (*G* matrix) within all families combined.** (a) Within the original 26 PX maternal families we observe a moderate relationship peak around 0, with a larger peak around 0.25, and a small peak around 0.5. (b) Within the corrected 26 PX maternal families, we observe the disappearance of the peak at 0 relationship. (c) Within the assigned 21 paternal families we observe a main relationship peak around 0.25, smaller peak around 0.5, and no relationship around 0.


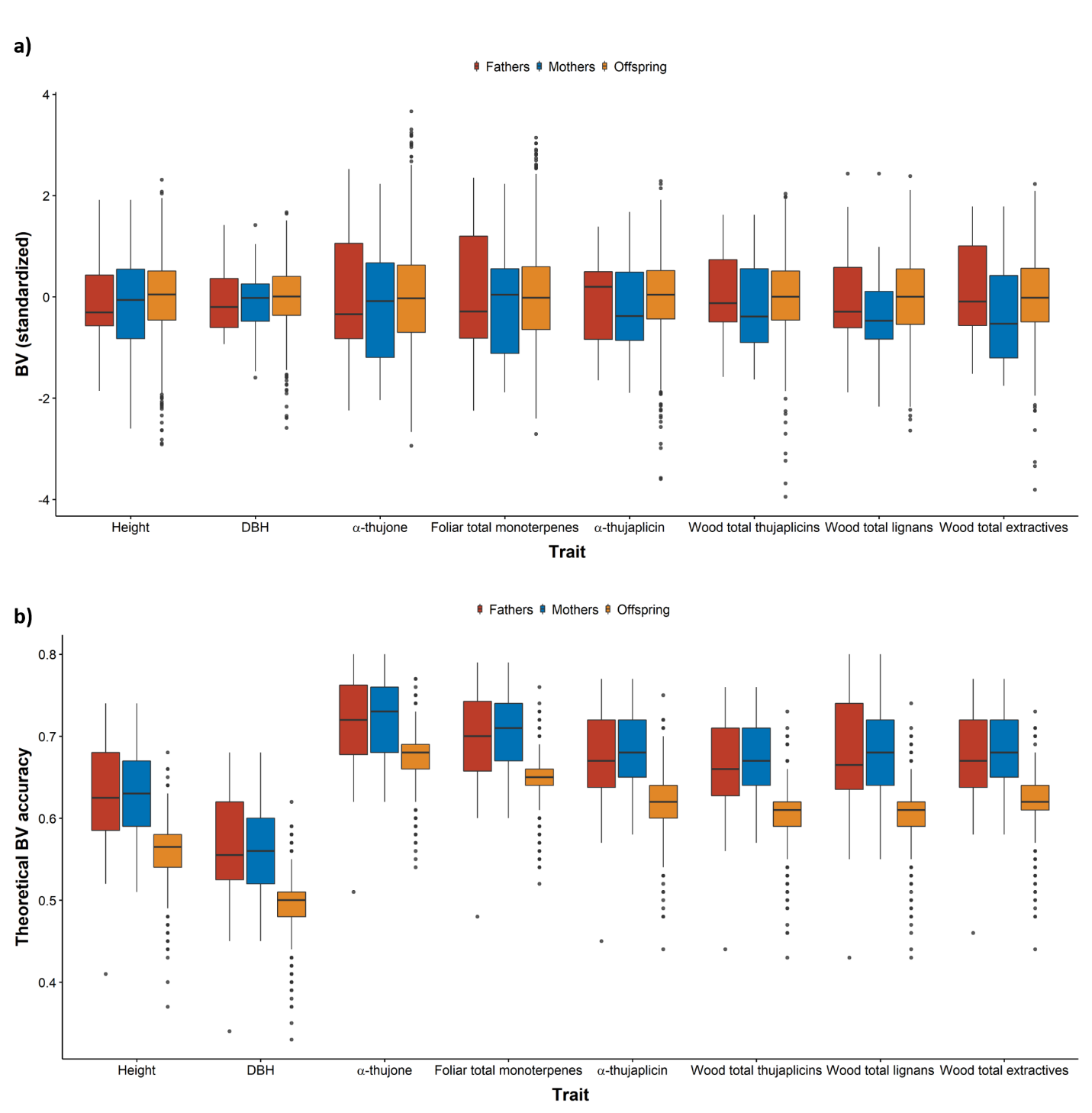
 **FIGURE S5** **Box plot of estimated breeding values and their theoretical accuracies for mothers, fathers and offspring using GBLUP-A for all traits.** (a) Estimated breeding values (BVs). BVs were standardized to remove the scale effect and include all traits in one plot. (b) BV theoretical accuracy ($\hat{r})$.
